# Using light to shape chemical gradients for parallel and automated analysis of chemotaxis

**DOI:** 10.15252/msb.20156027

**Published:** 2015-04-23

**Authors:** Sean R Collins, Hee Won Yang, Kimberly M Bonger, Emmanuel G Guignet, Thomas J Wandless, Tobias Meyer

**Affiliations:** 1 Department of Chemical and Systems Biology, Stanford University School of MedicineStanford, CA, USA

**Keywords:** chemokinesis, chemotaxis, Galphai, gradients, uncaging

## Abstract

Numerous molecular components have been identified that regulate the directed migration of eukaryotic cells toward sources of chemoattractant. However, how the components of this system are wired together to coordinate multiple aspects of the response, such as directionality, speed, and sensitivity to stimulus, remains poorly understood. Here we developed a method to shape chemoattractant gradients optically and analyze cellular chemotaxis responses of hundreds of living cells per well in 96-well format by measuring speed changes and directional accuracy. We then systematically characterized migration and chemotaxis phenotypes for 285 siRNA perturbations. A key finding was that the G-protein G_i_α subunit selectively controls the direction of migration while the receptor and Gβ subunit proportionally control both speed and direction. Furthermore, we demonstrate that neutrophils chemotax persistently in response to gradients of fMLF but only transiently in response to gradients of ATP. The method we introduce is applicable for diverse chemical cues and systematic perturbations, can be used to measure multiple cell migration and signaling parameters, and is compatible with low- and high-resolution fluorescence microscopy.

## Introduction

Many types of cells respond to spatial gradients of chemical cues (Segall, 1993; Ashe & Briscoe, 2006; Iglesias & Devreotes, 2008; Vladimirov & Sourjik, 2009; Swaney *et al*, 2010; Tojima *et al*, 2011). In chemotaxis, cells direct their movement toward the highest concentration of an attractant. Eukaryotic cells such as human neutrophils directly sense and respond to spatial concentration differences in chemoattractant, in contrast to bacteria that sense only temporal changes in attractant levels to chemotax (Iglesias & Devreotes, 2008; Vladimirov & Sourjik, 2009; Swaney *et al*, 2010). Despite many years of research, the molecular mechanisms responsible for spatial sensing have remained elusive. One challenge in the field has been that the response of cells to chemoattractant stimulation is complex and multifaceted. Cells respond by increasing their motility (chemokinesis), orienting their movement according to the gradient, and modulating adhesion dynamics, while additionally activating other pathways that may be unrelated to chemotaxis (Iglesias & Devreotes, 2008; Swaney *et al*, 2010). Furthermore, many implicated pathways and components are important for more than one aspect of the cell's migration response.

Existing assays for studying eukaryotic chemotaxis typically allow either high-throughput analysis with only indirect measurement of cell behavior or direct visualization of cells responding to gradients in one-at-a-time experiments (Zigmond *et al*, 2001; Pujic *et al*, 2009). Here we present a technique that combines high-throughput, automated, and systematic analysis with direct live-cell imaging. This approach has several advantages. Systematic, high-throughput experimentation allows measurement of a large number of perturbations using standardized conditions. Live-cell imaging allows measurement of multiple aspects of cell behavior (i.e. cell speed, direction, and signaling activities). In combination, it is possible to distinguish perturbations that affect multiple components of the behavior from those that are specific for one.

## Results

### Optical generation and control of chemical gradients

**Figure 1:**
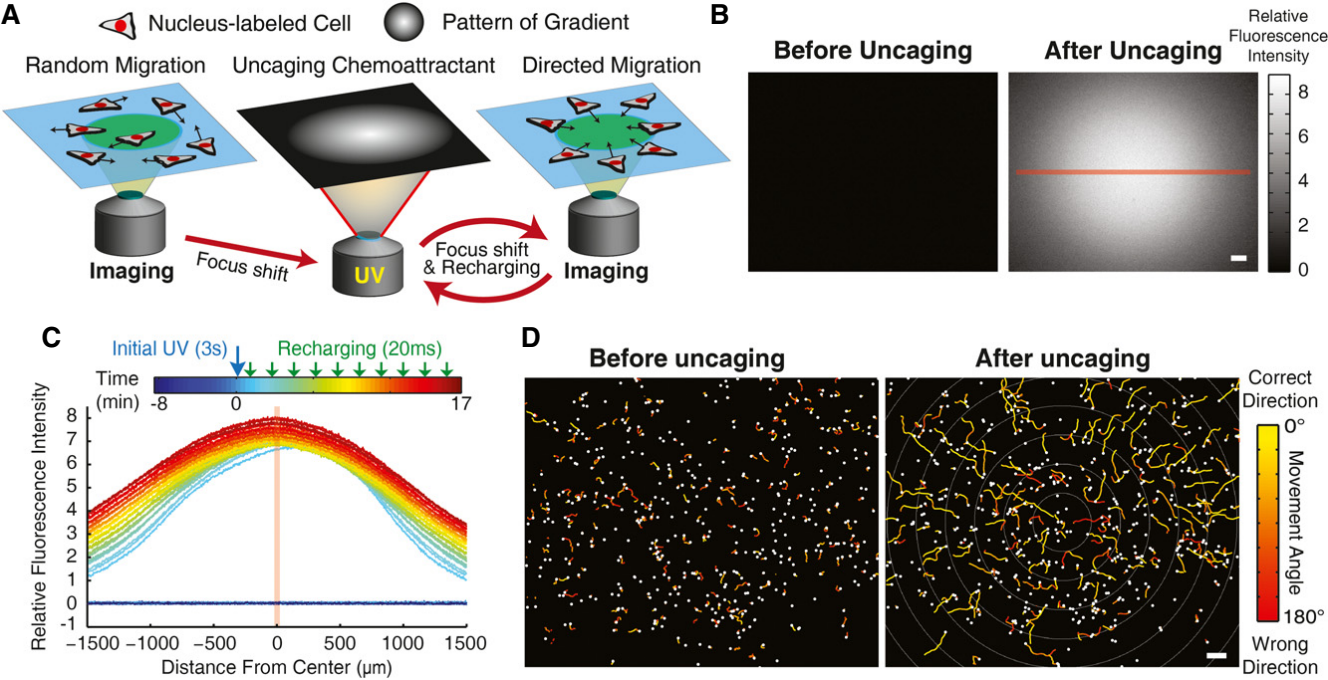
System for parallel and automated gradient generation and live-cell imaging of chemotaxis

To dissect the complex chemotactic response of individual cells to various stimuli, we developed a method to precisely control chemoattractant gradients in space and time. This was achieved by using a chemically caged derivative of a chemoattractant combined with automated control of the uncaging light (Fig1A).

Schematic of strategy for time-lapse live-cell imaging combined with automated manipulation of chemoattractant gradients using ultraviolet (UV) uncaging of a caged derivative of a chemoattractant. In between designated time points, a spatial gradient of UV light is produced with an out-of-focus objective to initiate or recharge the gradient. Recharging pulses were small periodic pulses intended to offset the effects of gradient dissipation by diffusion.

Images of spatial gradients of fluorescein before and after UV uncaging of CMNB-fluorescein. The left image was acquired 8 min and 41 s before the initial uncaging, and the right image was acquired 5 min and 18 s after the initial uncaging. The scale bar indicates a length of 100 μm.

Time course of the intensity profile of the fluorescein gradient cross-section indicated with the red line in (B).

Tracks of the movement of PLB-985 cells for 5-min intervals before (ending 37 s before uncaging) and after gradient generation (ending 17 min and 35 s after initial gradient generation) using a caged chemoattractant (Nv-fMLF) and the uncaging strategy described in (A). The tracks are overlaid on top of the images of the last time point in each time interval. Color indicates the direction of movement toward (yellow) or away from (red) the gradient center. The scale bar indicates a length of 100 μm.

Source data are available online for this figure.

We first calibrated our system using a caged derivative of fluorescein to monitor the generated gradients. We embedded the caged molecule in agarose gels to eliminate convection. Indeed, application of a spatial gradient of uncaging light generated a chemical gradient (Fig1B). These gradients corresponded to concentration changes of 2–5% over a distance of ∼30 μm, the approximate length of a human neutrophil. While a single uncaging pulse yielded a gradient that gradually dissipated by diffusion, we reasoned that small, periodic “recharging” pulses might be able to offset this effect. Empirical analysis of gradient diffusion combined with simulation of multiple pulse protocols (an initial pulse followed by periodic smaller recharging pulses) allowed us to devise a strategy for maintaining a relatively stable spatial gradient over timescales of tens of minutes. We verified the strategy with control measurements using caged fluorescein (Fig1C).

We next verified that this strategy can be combined with live cells migrating under agarose using a caged chemotactic ligand. The agarose above the cells provides a confined environment that may more closely reflect a neutrophil's environment *in vivo* (Friedl & Weigelin, 2008; Renkawitz & Sixt, 2010). We used differentiated PLB-985 cells as a model for human neutrophils (Tucker *et al*, 1987; Servant *et al*, 1999) and developed a protocol to polymerize gels of low-melting-temperature agarose in 96-well format on top of the live cells. Cells continued to migrate efficiently under the agarose gels. We next synthesized an N-nitroveratryl derivative (Nv-fMLF) of the classic chemoattractant N-formyl-methionine-leucine-phenylalanine (fMLF) (Pirrung *et al*, 2000). Consistent with its initial characterization (Pirrung *et al*, 2000), Nv-fMLF had little biological activity toward PLB-985 cells. However, when we combined our gradient uncaging strategy with Nv-fMLF, PLB-985 cells responded with a robust chemotaxis response (Fig1D; Supplementary Movie S1).

### Multi-parametric quantification of directed cell migration

**Figure 2:**
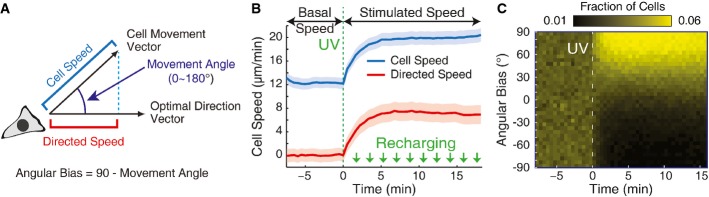
Quantification of motility and chemotaxis

A main goal of our approach was to quantify the key parameters of chemotactic movement from a single experiment. By tracking cells from frame to frame, we measured cell movement vectors that allowed us to measure cell speed and direction. To quantify cell direction, we measured the angle between the cell movement vector and an optimal direction vector pointed toward the gradient center (Fig2A). We computed a related “angular bias” parameter (90 minus the movement angle), such that random migration will give a mean angular bias value of zero, and a value of 90 indicates maximal directionality. The projection of the cell movement vector onto the optimal direction provides an alternate measure of chemotaxis (directed speed), with units of distance over time.

Scheme for quantifying speed and direction of movement. Cell speed is measured as the distance moved between consecutive images, divided by the time elapsed in between. A movement angle is measured between the cell's movement vector and a vector pointing toward the gradient center (the optimal direction vector). An angular bias parameter is computed as 90 minus the movement angle. Directed speed is measured as the component of cell velocity in the direction of the gradient center.

Measurement of mean cell speed and directed speed as a function of time, for PLB-985 cells using the experimental scheme described in Fig1. Initial gradient generation is marked by the dotted green line. Periodic smaller gradient recharging pulses are indicated by green arrows. The shaded region indicates the mean value plus or minus the standard error of the mean (*n *= 96 wells).

Heatmap of cell directionality as a function of time. Color indicates the fraction of individual cells moving in a given direction at each point in time. This data is from the same experiment as in (B).

Source data are available online for this figure.

We verified the chemotactic responses by measuring cell speed, directed speed, and angular bias as a function of time relative to gradient generation. We found that rapidly after gradient generation, cells sped up markedly (Fig2B) and gained a clear directional bias toward the center of the gradient (Fig2B and C). After about 5 min, the chemotaxis response hit a steady state and was approximately constant for the rest of the experiment. Furthermore, these chemotactic responses increased in a dose-dependent manner with attractant concentration (Supplementary Fig S1).

**Figure 3:**
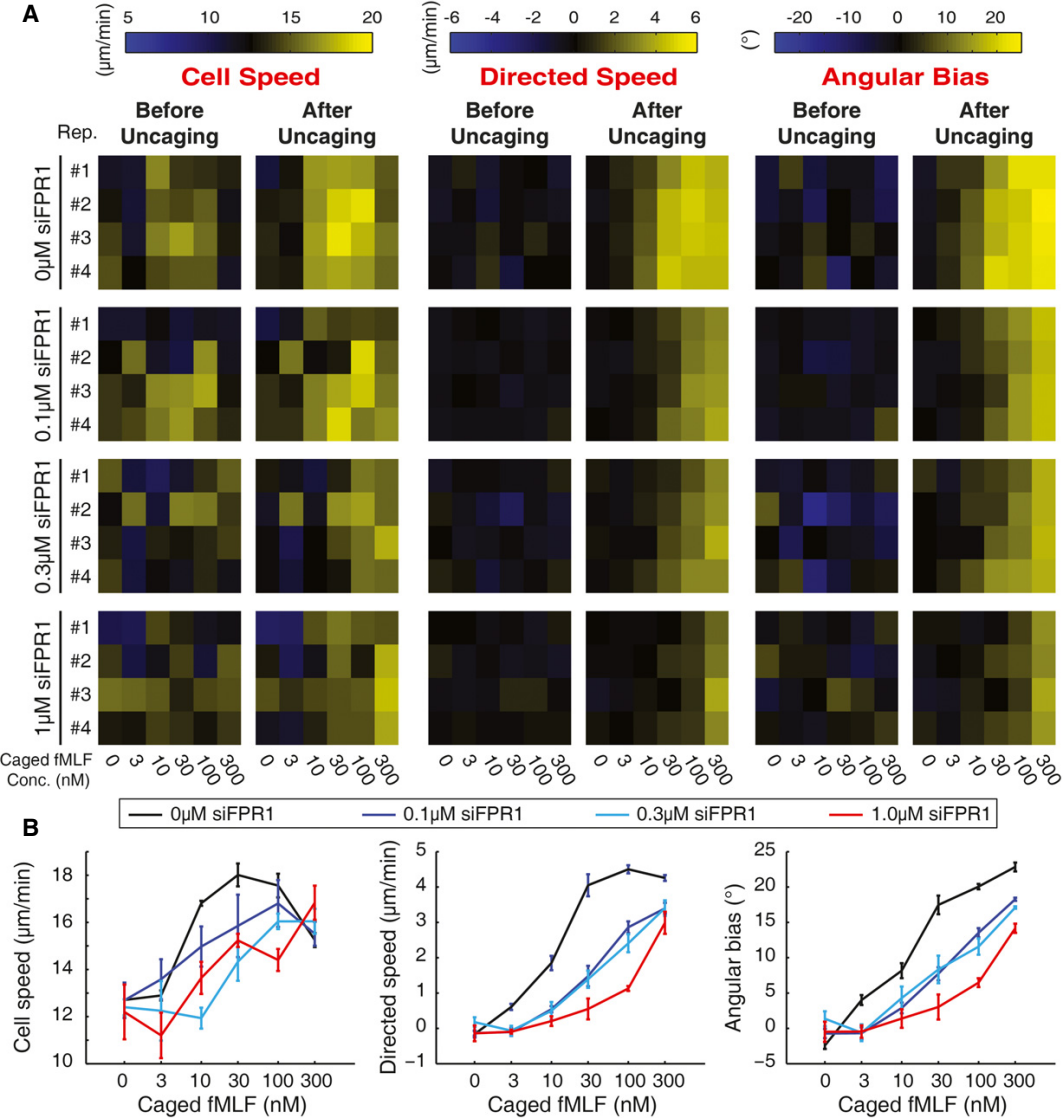
Reproducible detection of differences in chemotactic efficiency

To benchmark its use for quantitative phenotyping and high-throughput screening, we tested whether the assay reproducibly detects defects in chemotaxis of varying strength. To this end, we measured cell movement and direction in parallel under diverse siRNA and chemoattractant conditions. First, we treated cells with either 0, 0.1, 0.3, or 1.0 μM siRNA targeting the formyl peptide receptor (FPR1) (knockdown efficiency of FPR1 in Supplementary Fig S2). Additionally, we used six different chemoattractant gradient amplitude conditions (0, 3, 10, 30, 100, or 300 nM Nv-fMLF) in a single 96-well plate. Our analysis showed clear dose–response relationships for each siRNA treatment condition, with increasing concentrations of Nv-fMLF inducing increasingly strong chemotactic responses (Fig3). Importantly, we also measured reproducible differences between the siRNA treatments for each chemotaxis parameter (Fig3). Intermediate concentrations of siRNA gave intermediate phenotype strengths, whereas 1.0 μM siRNA resulted in reproducibly stronger chemotactic defects.

Heatmaps indicate the mean value of cell speed (left), directed speed (center), or angular bias (right) for individual wells with the indicated concentration of caged fMLF and PLB-985 cells treated with the indicated concentration of siRNA targeting the formyl peptide receptor (FPR1).

Dose–response curves computed using the data from (A) for each parameter and each siRNA condition. Error bars indicate the standard error of the mean over measurements of four independent wells.

Source data are available online for this figure.

### Parallel and automated analysis of chemotaxis phenotypes for gene perturbations

**Figure 4:**
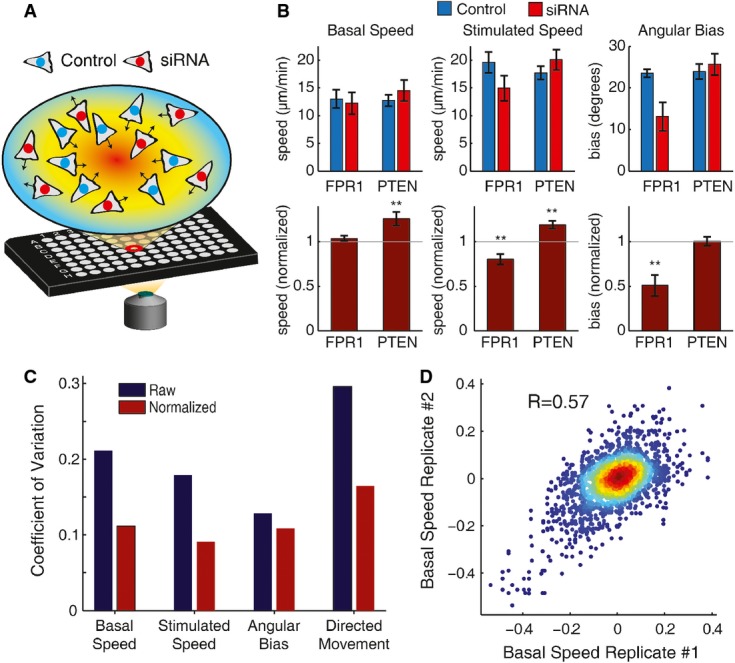
Normalization using an in-well control improves signal-to-noise

We next applied our assay for high-throughput characterization of chemotaxis and motility phenotypes for gene perturbations. High-throughput characterization and screening measurements are especially sensitive to experimental noise, as significant changes need to be identified despite stochastic variations in conditions and potential systematic errors among large numbers of samples. To minimize these types of errors, we placed two cell populations into each well: an siRNA-treated experimental cell sample labeled with one marker (e.g. a histone H2B-mCherry fusion protein) and a second cell sample treated with a control siRNA labeled with a different marker (e.g., a histone H2B-mTurquoise fusion protein) (Fig4A). We used the same control siRNA-treated cell population as a control in every well to correct for day-to-day and well-to-well variability in experimental conditions.

Schematic of chemotaxis assay in 96-well format using in-well control cell populations.

Above, measured cell movement parameters for the indicated siRNA conditions (red) and in-well controls (blue). Error bars represent the standard error of the mean over independent wells (*n *= 3). Below, relative cell movement parameters for the indicated siRNA conditions after normalization using the in-well controls for the same wells as above. Two-sided *P*-values were computed by fitting the empirical error distribution from the high-throughput experiments (see Materials and Methods). **P-values < 0.001 (exact *P*-values were *P *= 0.4 for FPR1 basal speed, *P *= 2 × 10^−8^ for PTEN basal speed, *P *= 2 × 10^−16^ for FPR1 stimulated speed, *P *= 1 × 10^−5^ for PTEN stimulated speed, *P *= 8 × 10^−9^ for FPR1 angular bias, and *P *= 0.9 for PTEN angular bias). Histograms of single cell data for instantaneous speed and directionality measurements for the siRNA and corresponding control cell populations are included in Supplementary Fig S3. All experiments in this figure used PLB-985 cells.

The coefficient of variation with (red) or without (blue) normalization using the in-well controls for the indicated cell movement parameters over 1,202 independent wells encompassing 285 different siRNA conditions.

Density-colored scatter plot of normalized basal speed phenotype scores (computed as the normalized basal speed measurement minus 1) from pairs of independent wells with identical siRNA conditions (including data for 285 different siRNA conditions). The Pearson's correlation for the scatter plot (0.57) is indicated. Corresponding scatter plots for other parameters are included in Supplementary Fig S4. The full set of individual replicate measurements for all parameters are included in Supplementary Dataset S1.

Source data are available online for this figure.

We applied our strategy systematically to 285 different siRNA pools in an arrayed one-condition-per-well format (the results of which are described below), introducing the siRNAs into PLB-985 cells using a previously described 96-well electroporation device (Guignet & Meyer, 2008). Target genes were selected based on the previously described connections to cell motility and chemotaxis, or for novel candidates, based on the presence of signaling domains and gene expression in neutrophils. Each siRNA condition was measured in at least three independent experiments so that experimental noise could be accurately estimated.

For gene perturbations known to impact chemotaxis and cell motility, we found clear differences in the behavior of experimental cells compared to the in-well controls. For example, as expected, treatment with siRNA targeting FPR1 caused no difference in cell speed before generation of the chemoattractant gradient, but caused a clear reduction both in cell speed in response to the gradient and in the directional accuracy of the cells (Fig4B; Supplementary Fig S3). In contrast, knockdown of PTEN, a known inhibitor of cell migration (Tamura *et al*, 1998), resulted in increased cell speed both before and after gradient generation, with no effect on the directional accuracy of the cells (Fig4B; Supplementary Fig S3). Thus, we can detect distinct phenotypes for siRNA perturbations relative to the internal controls.

Additionally, we verified that the internal control did reduce the measurement variability between wells. For each speed-related phenotype, we found that normalization using the internal control decreased the coefficient of variation approximately twofold (Fig4C). Furthermore, over the entire set of siRNA conditions, we found that the normalized scores were strongly correlated for replicate measurements from different experiments (Fig4D; Supplementary Fig S4; Supplementary Dataset S1).

**Figure 5:**
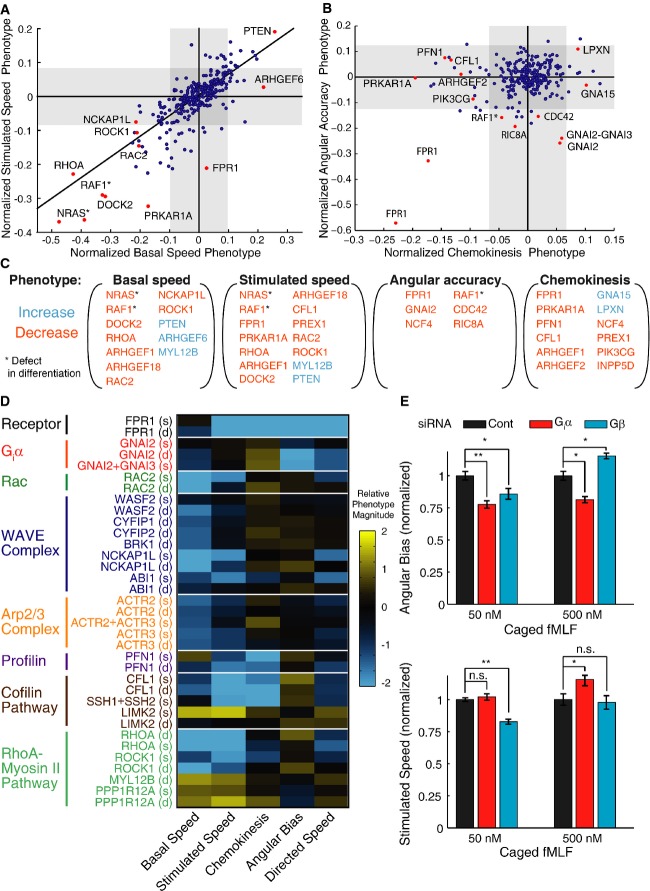
Systematic analysis of cell motility and chemotaxis phenotypes for siRNA perturbations

We analyzed our systematic chemotaxis experiments with the aim to distinguish different classes of gene perturbation phenotypes. In particular, we determined which gene perturbations affect motility in general before and after stimulation, which gene perturbations affect only the stimulated increase in speed, and which ones selectively regulate directional accuracy. To this end, we measured five phenotype scores for different aspects of cell behavior: basal speed (before the gradient), stimulated speed (in the presence of the gradient), chemokinesis (relative increase in speed in response to stimulation), angular bias (a measure of directional accuracy), and directed movement (the component of cell speed in the direction of the gradient). We found multiple overlapping and distinct gene perturbations affecting each phenotype (Fig5A–D, see Supplementary Dataset S2 for a full table of results), and we were interested in both general trends relating different phenotypes and the specific genes responsible for each aspect of behavior.

Scatter plot comparing mean phenotype scores for basal and post-stimulus cell speed for each of 285 different siRNA conditions. Every point represents the average of at least three independent wells. The gray-shaded regions indicate 98% confidence intervals based on fitting of the empirical error distribution (see Materials and Methods). All experiments in this figure used PLB-985 cells. The full set of phenotype scores are included in Supplementary Dataset S2.

Scatter plot of mean phenotype scores for chemokinesis and angular accuracy. The full set of phenotype scores are included in Supplementary Dataset S2.

Summary of siRNA conditions with reproducible phenotypes for each of the indicated cell movement parameters (basal speed, stimulated speed, angular bias, and chemokinesis). The text color for each gene name indicates the direction of the phenotype (blue text indicates an increased value and orange text indicates a decreased value relative to unperturbed cells). The asterisks next to NRAS and RAF1 indicate that siRNAs targeting these genes caused defects in differentiation (see Supplementary Dataset S3 and Supplementary Fig S6), and so their effects may be indirect.

A heatmap of relative phenotype scores [here the phenotype scores have been scaled by width of the corresponding gray regions in (A and B)] for siRNA conditions targeting the indicated genes in the pathways or protein complexes indicated on the left.

Above, normalized mean angular bias measurements for cell populations treated with nontargeting control siRNA, a pool of siRNAs targeting G_i_α (GNAI2 and GNAI3), and a pool of siRNAs targeting Gβ (GNB1 and GNB2). Below, normalized mean post-stimulus cell speed values for the same samples. Error bars represent the standard error of the mean for measurements of eight independent wells. Two-sided *P*-values were computed using the Mann–Whitney *U*-test. *P-value < 0.05, **P-value < 0.001 (exact *P*-values were *P *= 3 × 10^−4^ for G_i_α angular bias at 50 nM, *P *= 5 × 10^−2^ for Gβ angular bias at 50 nM, *P *= 3 × 10^−3^ for G_i_α angular bias at 500 nM, *P *= 1 × 10^−3^ for Gβ angular bias at 500 nM, *P *= 0.2 for G_i_α stimulated speed at 50 nM, *P *= 2 × 10^−4^ for Gβ stimulated speed at 50 nM, *P *= 1 × 10^−2^ for G_i_α stimulated speed at 500 nM, and *P *= 0.6 for Gβ stimulated speed at 500 nM).

Source data are available online for this figure.

First, we observed a strong correlation between basal speed and stimulated speed phenotypes (Fig5A). This correlation is likely due to a number of genes that affect cell motility in general, whether the cell is in a gradient or not. We thus fit a trendline between these parameters and interpreted positions along this trendline as indicative of a general cell motility phenotype. Our chemokinesis phenotype score was then defined by the distance from this trendline. As we expected, knockdown of FPR1 gave a strong chemokinesis phenotype, but had little or no effect on basal speed (Fig5A, C and D).

### Functional specialization of pathways in the control of directed cell migration

We next determined whether specific genes or pathways selectively control different measured chemotaxis parameters. We sorted genes according to their phenotype (Fig5C), excluding cases where results were inconsistent between independent siRNA pools targeting the same gene. In general, when we had independent pools targeting the same gene, the results were highly correlated (Supplementary Fig S5). We found a number of expected genes affecting general motility, including RAC2, genes encoding components of the WAVE regulatory and ARP2/3 complexes that regulate actin nucleation (Stradal & Scita, 2006; Derivery & Gautreau, 2010), and components of the RhoA–myosin II pathway that regulate contractile force (Charest & Firtel, 2007) (Fig5C and D). However, we also found a number of more surprising results. For example, siRNAs targeting the actin monomer delivering protein profilin (PFN1), the actin-severing protein cofilin (CFL1), and the cofilin-activating phosphatase slingshot (SSH1 and SSH2) all caused marked chemokinesis defects, and affected chemokinesis more strongly than they affected basal motility (Fig5C and D). These results suggest that breakdown and recycling of the actin network (Bravo-Cordero *et al*, 2013) may be a key mechanism specifically regulating maximal speed.

We reasoned that a critical means to assess the regulation of directional accuracy is a comparison of measured phenotypes for chemokinesis and angular bias. These parameters report two key features of the cell's response to stimulation when a chemoattractant gradient is applied. One simple hypothesis would be that all perturbations have proportional effects on these two parameters. Such a result is expected if the two responses were intertwined, for example if cells steered by boosting their speed in the optimal direction. While proportional defects were observed for some genes, such as the formyl peptide receptor itself, we found a number of gene perturbations that uncoupled the two responses (Fig5B). Suppression of profilin, cofilin, and slingshot altered chemokinesis with little directional effect, again consistent with the hypothesis that rapid actin remodeling is critical for controlling maximal speed. In addition, the PKA regulatory subunit (PRKAR1A) and the RhoA-activating GEF-H1 (ARHGEF2) also had predominantly chemokinesis effects, suggesting that the associated signaling pathways primarily act to regulate speed rather than direction in response to chemoattractant.

A strikingly opposite result was that the siRNA pools targeting G_i_α (GNAI2 and GNAI3) strongly affected cell direction with little effect on chemokinesis. This result was surprising, as it differed markedly from the phenotype of knocking down the receptor FPR1 (Fig5B). However, we got the same result using both our diced pool targeting GNAI2, which is the major G_i_α isoform in neutrophils, and a commercial mixture of synthesized siRNA pools targeting GNAI2 and GNAI3 (Fig5B and D). A commercial siRNA pool targeting only GNAI2 had a similar, but weaker phenotype (Fig5D). We reasoned that an explanation for the difference between G_i_α and FPR1 phenotypes could be the specialization of the G_i_α and Gβγ complexes downstream of the receptor. To test this, we compared phenotypes for siRNAs targeting G_i_α and Gβ (Gβ had not been in our initial library) at two different doses of chemoattractant. Indeed, we found markedly different phenotypes for the two G-protein subunits. We found that at a low dose of chemoattractant (50 nM Nv-fMLF), knockdown of either G_i_α or Gβ caused a defect in directional sensing (Fig5E). However, only knockdown of Gβ caused a defect in cell speed in the gradient (Fig5E). With a high dose of chemoattractant (500 nM Nv-fMLF), both defects caused by the Gβ knockdown disappeared, but the direction phenotype for G_i_α remained. These results suggest that levels of Gβ are critical for sensitivity of the system—a small amount of Gβ is sufficient if the stimulus is strong, but for weak signals, the level of Gβ is critical for both speed and directional responses. In contrast, G_i_α may have a distinct role in regulating directional migration. Even with a high concentration of ligand, efficient processing of directional information is still strongly dependent on G_i_α levels. Our evidence for specialization of the G-protein subunits could in part be explained by a proposed pathway linking G_i_α to the PKCζ-containing PAR complex to control cell direction (Kamakura *et al*, 2013). However, this may not be the only role of G_i_α as our analysis of two siRNA pools targeting PKCζ (PRKCZ) did not show a directionality phenotype.

As a technical note, since our model system requires differentiation into a neutrophil-like state, we also assessed the effect of each siRNA pool on differentiation by measuring cell surface levels of the receptor FPR1 (Supplementary Dataset S3; Supplementary Fig S6). Importantly, we found only two of the tested genes, NRAS and RAF1, for which siRNA substantially inhibited differentiation (Supplementary Fig S6).

### Dynamic chemotactic responses to gradients of ATP

We next tested whether the same approach can also be applied to other caged derivatives of known or putative chemoattractants. We focused on ATP to compare and contrast the chemotactic response of neutrophils to fMLF and ATP. Like most other known chemoattractants for neutrophils, ATP is detected by a G_i_α-activating receptor (Meshki *et al*, 2006). Autocrine signaling using ATP has also been proposed to be an important part of the neutrophil chemotactic system (Chen *et al*, 2006). However, earlier results suggested that stimulation of neutrophils with ATP induces chemokinesis, but not directional chemotaxis (Chen *et al*, 2006; Isfort *et al*, 2011).

**Figure 6:**
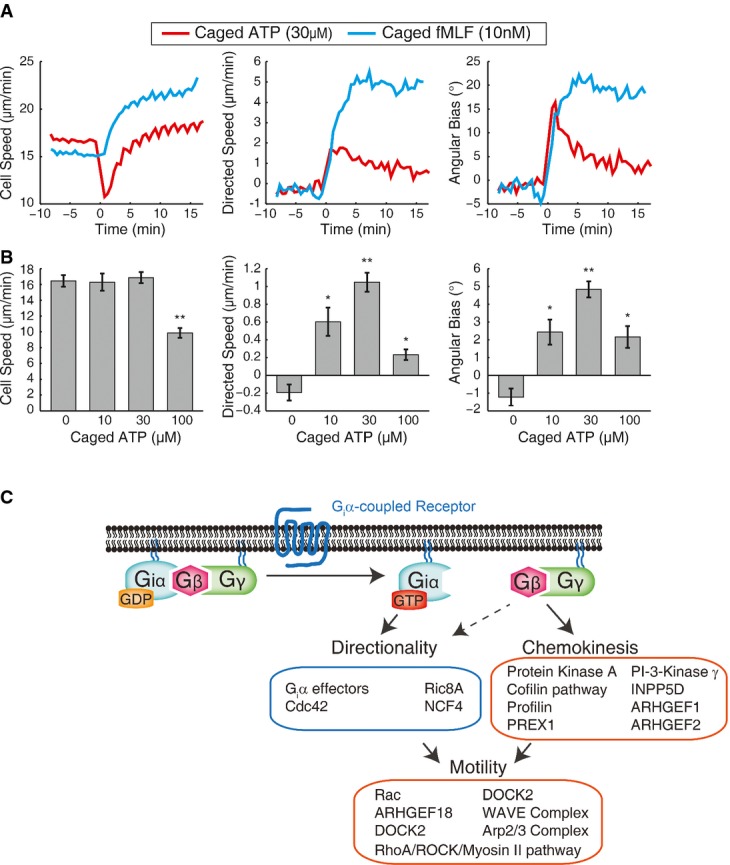
Contrasting chemotactic responses to fMLF and ATP

Using our strategy with the caged molecule NPE-ATP (Kaplan *et al*, 1978), we observed significant chemotaxis in response to an ATP gradient. Interestingly, however, the directional response to ATP was short-lived. While fMLF induced steady directional movement over tens of minutes, the directional response to ATP was initially strong but had largely disappeared after 5 min (Fig6A). The transient nature of the chemotactic response may explain why it was previously not observed, and highlights a strength of our approach to observe transient changes in responses and sensitivities. With the ability to generate gradients rapidly in a matter of seconds, rather than relying on slow diffusion-based strategies, our method allows visualization and measurement of both instantaneous responses and adaptive changes. In addition to the chemotactic response, we also observed a cell stopping response to high doses of ATP stimulation (Fig6A and B, Supplementary Fig S7; Supplementary Movie S2). This response was not observed for fMLF, suggesting that a different balance of downstream pathways is activated in response to ATP stimulation.

Plots of cell speed, directed speed, and angular bias as a function of time for PLB-985 cells responding to uncaging-induced gradients of fMLF (blue) and ATP (red). The gradients were generated at time 0. Each curve represents the mean of measurements from eight independent wells.

Bar graphs of mean cell speed, directed speed, and angular bias for PLB-985 cells in gradients of ATP generated by uncaging using the indicated concentrations of caged ATP. Data were averaged over ˜17 min in the gradient. Error bars represent the standard error of the mean for eight independent wells. Two-sided *P*-values were computed using the Mann–Whitney *U*-test. *P-value < 0.05, **P-value < 0.001 (exact *P*-values were *P *= 0.8 for cell speed at 10 μM, *P *= 0.4 for cell speed at 30 μM, *P *= 1 × 10^−4^ for cell speed at 100 μM, *P *= 1 × 10^−3^ for directed speed at 10 μM, *P *= 1 × 10^−4^ for directed speed at 30 μM, *P *= 4 × 10^−3^ for directed speed at 100 μM, *P *= 2 × 10^−3^ for angular bias at 10 μM, *P *= 1 × 10^−4^ for angular bias at 30 μM, and *P *= 1 × 10^−3^ for angular bias at 100 μM).

Schematic model of specialization of components for basic motility, increasing speed (chemokinesis), and refining directionality upon stimulation of G_i_α-coupled receptor with a gradient of chemoattractant based on our results using systematic siRNA-mediated perturbations in PLB-985 cells and uncaging of Nv-fMLF. Groups of genes and pathways are shown based on the phenotypic parameters primarily affected by siRNA perturbations.

Source data are available online for this figure.

## Discussion

Our study introduces an integrated system to explore the architecture of cellular pathways regulating chemotaxis and migration. In particular, our approach combines automated and parallel manipulation of chemoattractant gradients with live-cell microscopy, allowing measurement of cell movement parameters (basal and stimulated velocity as well as chemotaxis parameters) for hundreds of cells per well in a single experiment. The rapid optical induction and maintenance over time of gradients allows measurement of both acute and adaptive responses. A 96-well plate format and use of in-well controls distinguished by differential color labels allows systematic and quantitative comparison of siRNA perturbed cells to a reference cell population.

By applying this strategy to a focused set of migration-related siRNAs, we were able to organize targeted genes into different phenotypic classes, most notably distinguishing signaling and cytoskeletal components responsible for general cell motility, signal-induced regulation of motility (chemokinesis), and directionality. Our results suggest a modular organization of chemotaxis responses, in which a core system mediates cell motility in the presence or absence of attractant stimulation, but this system receives inputs from additional specialized pathways to boost motility and refine direction in response to gradients of chemoattractant (Fig6C). The motility phenotypes we observe for Rac2 and components of the Arp2/3 and WAVE complexes which drive actin polymerization at the cell front, as well as RhoA and ROCK1 that regulate myosin contractility at the cell back, are consistent with roles for these components as central pieces of the core motility machinery.

Interestingly, we found distinct sets of genes regulating chemokinesis and directionality. The strongest directionality-specific phenotype was that of the G_i_α heterotrimeric G-protein subunit immediately downstream of the receptor. Knockdown of G_i_α gave a strong directionality phenotype with a minimal effect on chemokinesis. In contrast, the beta subunit, which is known to activate phosphoinositide 3-kinase (PI3K) (Hawkins *et al*, 2010), as well as the receptor FPR1 itself showed coupled effects on both direction and chemokinesis. This distinct behavior for G_i_α was evident over a range of chemoattractant concentrations and using a number of different siRNAs to target G_i_α versus the receptor. Our results support a model in which the G-protein subunits play specialized roles downstream of the receptor, with Gβγ driving signal amplification and promoting sensitivity of the response through effectors such as PI3K, and G_i_α acting through other pathways to refine directionality.

In contrast to the G-proteins and the receptor, we found that knockdowns of other genes including cofilin and its upstream regulator the slingshot phosphatase, as well as profilin, the RhoA activator GEF-H1 (ARHGEF2), and the PKA regulatory subunit PRKAR1A, each caused primarily defects in chemokinesis but not directionality. Our identification of the cofilin pathway as an important regulator of chemokinesis was notable, as this pathway has been characterized as controlling directionality in cancer cell migration (Mouneimne *et al*, 2004), but does not appear to regulate directionality in *Dictyostelium discoideum* (Kölsch *et al*, 2008). Although localized cofilin activity is capable of steering protrusions, our results indicate that the extreme directional accuracy of dedicated chemotactic cells such as neutrophils is achieved through a G_i_α-directed mechanism, while the cofilin pathway acts in parallel primarily to boost motility and speed, perhaps also integrating mechanical information (Hayakawa *et al*, 2011). Our phenotypes for profilin and the slingshot–cofilin pathway are consistent with a regulated and rate-limiting role for the turnover of the actin filament network in controlling cell speed.

Our study gives insights into the complex regulation of the Rho family GTPases during cell migration. Neutrophils express a panel of GEF and GAP proteins implicated in the regulation of Rho family GTPases. Notably, we found strong phenotypes for three different GEFs that regulate RhoA (Cook *et al*, 2014). However, knockdowns of each of these GEFs fell into a different phenotypic class. Knockdown of p114-RhoGEF (ARHGEF18) affected general motility, but not chemokinesis, knockdown of GEF-H1 caused a strong chemokinesis defect, with little effect on basal motility, and knockdown of p115-RhoGEF affected both basal motility and chemokinesis (Fig5C, Supplementary Dataset S2). Our surprising observation that each of these GEFs plays an important role raises the interesting question how their different activation and feedback connections allow them to shape the activity of RhoA in space and time during chemotaxis.

Additionally, time-lapse imaging and rapid gradient manipulation allow characterization of temporal dynamics in chemotaxis responses. Although previous studies only observed chemokinesis (and not chemotaxis) in response to ATP, we observed a strong, but short-lived chemotactic response. The short-lived nature of the response likely made it undetectable with previously used methods, which typically rely on slow diffusion-based methods to establish attractant gradients. Our results also provide an explanation to a puzzle: ATP activates the P2Y_2_ receptor that activates G_i_α in neutrophils (Meshki *et al*, 2006), and activation of G_i_α drives chemotaxis downstream of other receptors even in heterologous systems (Neptune & Bourne, 1997). Nonetheless, neutrophil chemotaxis to ATP had not been observed to our knowledge before this work. Our results indicate that ATP does induce chemotaxis, but additional pathways activated by ATP may dampen the chemotactic response after a short time period. Thus, our strategy is suitable to investigate adaptive mechanisms that modulate or abolish chemotaxis after an initial response to stimulus.

Since the method can be used with other caged molecules, it will allow for comparative analyses of different chemoattractants and their corresponding signaling pathways. For example, the method could be used to systematically explore the hierarchy of leukocyte “end target” versus “intermediary” chemoattractants which can differ in having broad cellular responses or more selective roles (Heit *et al*, 2002, 2008; Ye, 2010). Finally, as the method uses live-cell microscopy, it can readily be adapted to other types and modes of imaging. It is compatible with imaging of fluorescent biosensors to monitor signaling events during migration and chemotaxis, and with small modifications, the same method can be used with other imaging modes such as confocal, total internal reflection, or super-resolution microscopy.

## Materials and Methods

### Cell culture

PLB-985 cells were obtained as a gift from the laboratory of Dr. Orion Weiner. Stable cell lines expressing fusions of histone H2B to mTurquoise or mCherry were generated by lentiviral transduction, followed by fluorescence-activated cell sorting in the Stanford Shared FACS Facility. Cells were cultured in RPMI 1640 with HEPES and glutamine (Life Technologies, catalog # 22400) supplemented with 9% fetal bovine serum, penicillin (100 units/ml), streptomycin (100 μg/ml), and glutamine (0.29 mg/ml) (growth medium) in humidified incubators at 37°C in the presence of 5% CO_2_. Cell cultures were passaged two to three times per week, maintaining cell densities between 10^5^ and 10^6^ cells per ml. The cells were differentiated into a neutrophil-like state by culturing at an initial density of 2 × 10^5^ in growth medium supplemented with 1.3% DMSO (differentiation medium) for 6 days (Tucker *et al*, 1987; Servant *et al*, 1999).

### Constructs

Histone H2B fused to mTurquoise or mCherry was cloned into the CSII-EF lentiviral vector.

### Caged molecules

The N-nitroveratryl derivative (Nv-fMLF) of N-formyl-methionine-alanine-phenylalanine was synthesized according to the published protocol (Pirrung *et al*, 2000). CMNB-fluorescein (catalog # F-7103) and NPE-ATP (catalog # A-1048) were purchased from Life Technologies.

### Preparation of 96-well format under agarose cell migration conditions

A warm liquid agarose-media mixture was prepared from two solutions. First, low-melting-temperature agarose was fully solubilized and dissolved in Leibovitz-15 media at a concentration of 3% (weight to volume) by repeated microwaving for 5–10 s at a time interspersed with mixing by swirling. (Note: the properties of low-melting-temperature agarose vary widely between manufacturers and specifications. Using an agarose with appropriate properties (such as Affymetrix product # 32830) is critical!) The agarose mixture was placed in a water bath at 37°C. Separately, a solution of Leibovitz-15 media supplemented with 17% fetal bovine serum and twice the desired final concentration of caged chemoattractant was prepared and placed in the same water bath. Both solutions contained penicillin, streptomycin, and glutamine added at the same concentration as growth media. PLB-985 cells were diluted to a density of 2 × 10^5^ per ml in Leibovitz-15 medium. Five microliters of cell solution was then added to the center of each well of a glass-bottom 96-well plate (Greiner Bio-One), and cells were allowed to settle for about 5 min. The two warm solutions were then mixed thoroughly, and 195 μl of this warm liquid agarose solution was added by slow careful pipetting at the edge of each well on top of the cells. The plate was then loosely covered with aluminum foil to protect it from light, while allowing air circulation. The plate was left at room temperature for 40 min to allow the agarose to solidify. The plate was then sealed with an adhesive foil seal, using a roller and/or pen cap to make sure the seal was uniformly tight around the well and plate edges. The plate was left at room temperature for an additional 5 min and then moved to the preheated 37°C microscope environment chamber. The plate was allowed to equilibrate for 45 min at 37°C before starting the imaging experiment. (Note: cell speed varies with temperature, and therefore, full temperature equilibration is important for consistent results.)

### Time-lapse microscopy with chemoattractant uncaging

An ImageXpress 5000A (Axon Instruments/Molecular Devices) was programmed to image a 96-well plate, by imaging sequential groups of 12 or 16 wells simultaneously, and then proceeding to the next group until all wells had been imaged. An environment chamber was used to maintain a 37°C temperature, and wells were imaged at 4× magnification every 30 s (or ∼40 s when faster imaging was not possible) in the desired imaging channels (typically CFP and RFP filter sets). A full experiment consisted of 50 frames (17 frames before gradient generation and 33 frames after). The initial gradient was generated with a 2.5 s exposure of ultraviolet (∼350 nm) light using a DAPI filter set (D360/40X excitation filter) and a 20× objective focused 1.5 mm below the bottom of the well. Using out-of-focus illumination generated a smooth approximately radially symmetric pattern of light. After the initial uncaging, the gradient of chemoattractant was maintained with 20-ms exposures every three frames using the same objective and focus position as for the initial uncaging.

### Image processing

Our image processing workflow included background subtraction, automated cell segmentation, and cell tracking. All processing was done using custom Matlab software. A smooth, locally estimated background was computed for each 1,024 × 1,280 image by computing the 80^th^ percentile pixel intensity for blocks of 80 × 80 μm (32 × 32 pixels), followed by bilinear interpolation. The background was subtracted from the image prior to cell segmentation. Cells were segmented by applying a manually selected intensity threshold (a single threshold was used for all wells and all time points), followed by application of the watershed algorithm to separate immediately adjacent cells. For each detected cell, its centroid and area were computed. Objects with areas > 75 pixels (∼470 square microns) or ≤ 5 pixels (∼31 square microns, though objects would typically need to be substantially smaller than this to be detected in only five pixels) were excluded from further analysis. Cells were tracked from frame to frame by identifying the nearest neighbor in the latter frame for each cell in the prior frame (forward nearest neighbor) and the nearest neighbor in the prior frame for each cell in the latter frame (backward nearest neighbor). Only unambiguous matches where both the forward and backward nearest neighbor computation gave the same assignment were used for further analysis. Additionally, for each cell in each frame, the distance to its nearest neighbor in the same frame was computed. Based on empirical analysis, a threshold distance of 40 μm (16 pixels) was applied such that if the nearest neighbor in both the prior and latter frames was within the threshold distance, that step was excluded for further analysis. This filter reduced the effects of occasional tracking errors. Cell steps from frame to frame were linked to generate cell trajectories.

### Statistics of cell movement

From the cell trajectories, we computed statistics to measure multiple aspects of cell movement. All statistics were computed using custom Matlab software.

First, we applied a filter to remove inappropriate cells from analysis. We observed that in every experiment, a small fraction of cells never moved. These nonmoving cells may include dead or unhealthy cells, as well as cells that did not fully differentiate into a neutrophil-like state. Indeed, when we performed similar experiments with undifferentiated or partially (< 6 days) differentiated cells, the proportion of nonmoving cells was substantially increased. For this reason, we excluded from analysis all cell trajectories for which the cell did not move more than two pixels from its initial location. Additionally, for every siRNA condition, we used an independent cytometry-based assay (see Supplementary Fig S6 and ‘Measuring cell differentiation’ section in the Materials and Methods) to assess the efficiency of differentiation.

From the cell tracks computed using the above-described method, we computed statistics of cell movement. For every tracked cell step between adjacent frames, we computed a movement vector from the cell centroid position in the prior frame to the cell centroid position in the latter frame. We computed the distance moved as the length of this movement vector. We computed the angle of movement as the angle between the movement vector and an optimal direction unit vector from the cell centroid position in the prior frame pointing toward the center of the image (which also represents the center of the chemoattractant gradient). An angle of 0 thus represented movement toward the center of the gradient, and an angle of 180° represented movement directly away from the center of the gradient. In order to avoid noise in angle measurements for nonmoving cells (measurements for these cells would only reflect noise in cell localization), we applied a minimum distance moved threshold of 10 μm (four pixels) for angle measurements. We computed a directed movement length as the dot product between the movement vector and the optimal direction unit vector.

From the aggregated cell step measurements, we computed mean cell speed (separately for the time period before and after gradient generation) as the mean of the movement distances divided by the corresponding time intervals. We computed mean directed movement as the mean of the directed movement distances divided by the corresponding time intervals. We computed angular bias as 90 minus the mean movement angle. Thus, an angular bias of zero corresponds to random direction relative to the gradient, an angular bias of 90 corresponds to maximal directionality toward the center of the gradient, and a negative angular bias corresponds to movement away from the center of the gradient. Additionally, as the local concentration of attractant is lower farther from the gradient center, we used empirical analysis to define a minimum and maximum distance from the gradient center (250 and 1,625 μm, respectively) for the inclusion of cell steps for computation of cell movement statistics in the gradient. In the region defined by these bounds, cell behavior was relatively homogeneous.

For 96-well experiments, normalized statistics and phenotype scores were also computed. Normalized statistics were computed using the statistics for experimental and internal control samples. We used robust linear regression (Matlab's robustfit function) to fit a trendline describing the experimental values as a function of the corresponding internal control values. Robust regression was used to minimize the effect of outliers, which typically result from strong siRNA phenotypes. We used this trendline to compute an expected value for each well, and then we computed the normalized value by dividing the experimental value by the expected value. This normalization based on the trendline is advantageous over the simple normalization of dividing by the internal control value because it allows for systematic day-to-day differences between control and experimental cells (e.g. on 1 day, all the control cells are systematically faster than the experimental cells), and it reduces potential over-correction due to noise in the control samples. In cases where the experimental values and control values were not positively correlated (Spearman rank correlation coefficient < 0.1 or a negative slope for the trendline), we normalized experimental values by dividing by the plate median instead of using the internal controls. This latter method typically applied for the angular bias measurements where well-to-well variation was often smaller than statistical noise. We then computed phenotype scores by subtracting 1 from the normalized values. We also computed a chemokinesis phenotype score from the basal speed (before the gradient) and stimulated speed (in the gradient) phenotype scores. To do this, we again used robust regression to fit a trendline describing the stimulated speed score as a function of the basal speed score. We used this trendline to compute an expected stimulated speed score, given a basal speed score. We then subtracted the observed minus the expected stimulated speed score (i.e. the distance above or below the trendline) to get a chemokinesis phenotype score.

### Error estimation for high-throughput results

Our high-throughput analysis of siRNA-based gene perturbations included at least three independent replicates of every perturbation. We used the differences between these replicate measurements to fit an experimental error model. In particular, given a probability distribution of error values, the distribution of differences between replicate measurements can be computed as the convolution of the error distribution with itself. We modeled the experimental error distribution for each phenotype score as a sum of two Gaussian distributions and fit the parameters using the observed distribution of differences between replicates using maximum likelihood estimation (Matlab's mle function). We used the resulting error model to compute a confidence interval within which the mean of three independent measurements for 98% of perturbations with zero true phenotype would fall. These confidence intervals are indicated by the gray regions in Fig5A and B. The same error model was used to compute two-sided *P*-values for the normalized movement statistics shown in Fig4B.

### siRNA electroporation

PLB-985 cells differentiated for 3 days were spun down and resuspended in extracellular buffer (5 mM KCl, 125 mM NaCl, 1.5 mM CaCl_2_, 1.5 mM MgCl_2_, 10 mM glucose, 20 mM HEPES, pH 7.4) at a density of 30 million cells per ml. The cell suspension was mixed with siRNAs to achieve a final concentration of 0.9 μM (Dharmacon siRNAs) or 14.5 ng/μl (diced pools of siRNA). siRNAs were then introduced into cells by parallel electroporation using a custom-built 96-well electroporation device (Guignet & Meyer, 2008) in 10-μl volumes with square-wave pulses of 115 V for 9 ms. Cells were then ejected into a 96-well plate with 200 μl of fresh differentiation medium (see ‘Cell culture’ above). After electroporation, additional differentiation medium was added to each well to bring the total well volumes to 310 μl. The cells were then left in differentiation medium (in 96-well plate format) for 3 more days to complete differentiation. To reduce the effects of evaporation and prevent plate edge effects, the spaces in between wells were filled with 175 μl of deionized water.

### Measuring cell differentiation

We measured differentiation of PLB-985 cells into a neutrophil-like state by measuring the levels of the formyl peptide receptor FPR1 on the cell surface. Relative levels of FPR1 were assessed by binding of N-formyl-norleucyl-leucyl-phenylalanyl-norleucyl-tyrosyl-lysine-fluorescein (FLPEP) (Life Technologies product number F1314), a fluorescent ligand of the receptor. Cells were mixed with ice cold media containing FLPEP to give a final concentration of 5 nM, placed on ice for 10 min, and analyzed by cytometry using a FACSCalibur cytometer (Becton, Dickinson, and Company).

### Data availability

The Matlab code used for the analyses is provided in the Supplementary Code and is also freely available for download on Github at https://github.com/srcollins/HT_Chemotaxis_Toolbox.

## Supporting Information

Supplementary Legends

Supplementary Code

Review Process File

Source Data for Figure 1C

Source Data for Figure 2B

Source Data for Figure 2C

Source Data for Figure 3A

Source Data for Figure 4B

Source Data for Figure 4C

Source Data for Figure 5D

Source Data for Figure 5E

Source Data for Figure 6A

Source Data for Figure 6B
