# Supplementary material for: Using light to shape chemical gradients for parallel and automated analysis of chemotaxis
Source: Mol Syst Biol. 2015 Apr 23;11(4):804. doi: 10.15252/msb.20156027 (PMC4422560; doi:10.15252/msb.20156027)
Supplement: Supplementary file 1 [file msb0011-0804-sd1.pdf]

Supplementary Figure 1

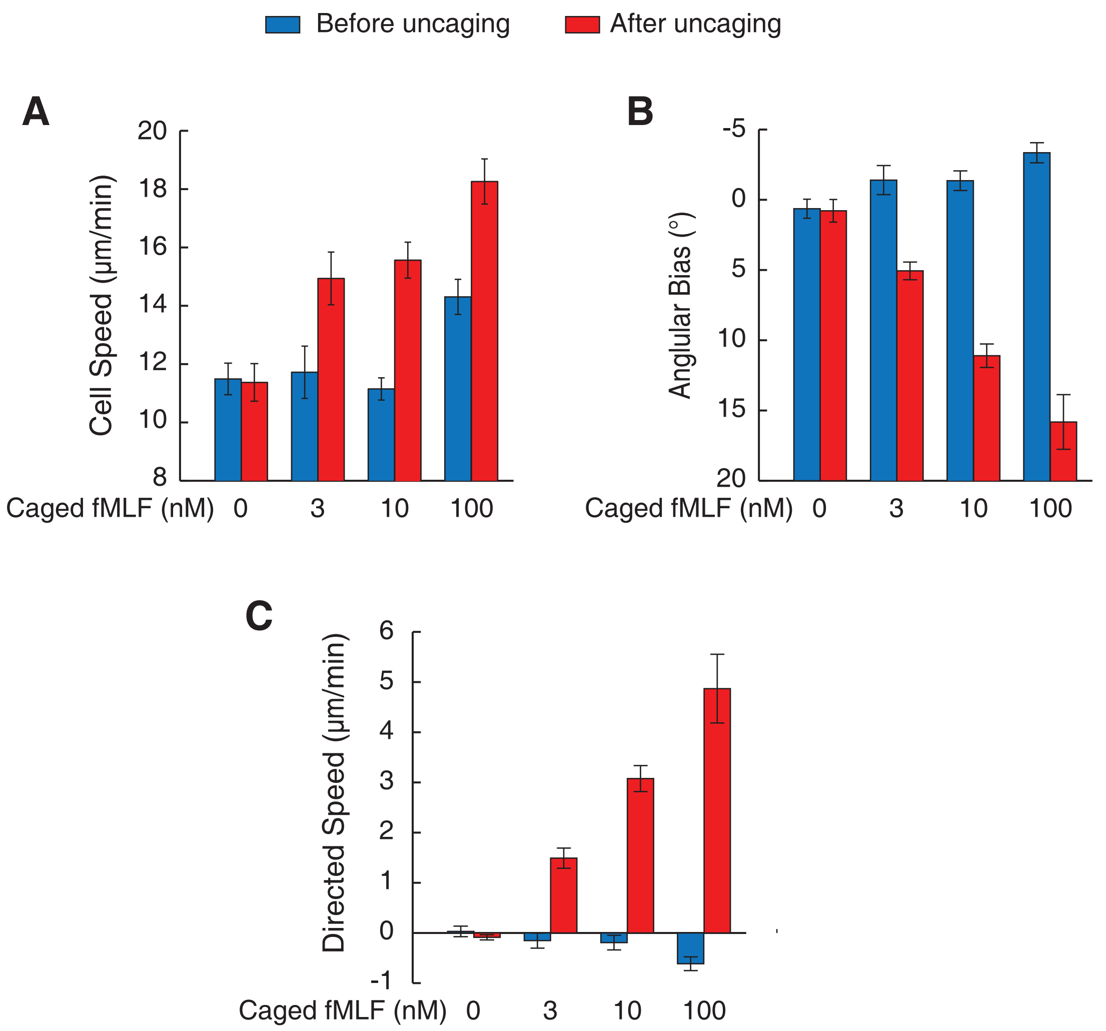

Supplementary Figure S1. Dose-dependent chemotactic responses to gradients of fMLF generated by uncaging.
A,B,C, Mean cell speed (A), angular bias (B), and directed speed (C) before (blue) and after (red) generation of gradients from the indicated concentrations of caged fMLF (Nv-fMLF).
