# Supplementary material for: Using light to shape chemical gradients for parallel and automated analysis of chemotaxis
Source: Mol Syst Biol. 2015 Apr 23;11(4):804. doi: 10.15252/msb.20156027 (PMC4422560; doi:10.15252/msb.20156027)
Supplement: Supplementary file 2 [file msb0011-0804-sd2.pdf]

Supplementary Figure 2

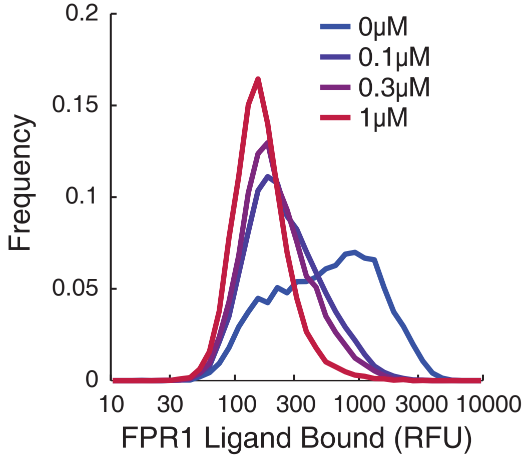

Supplementary Figure S2. Dose-dependent knockdown of FPR1 by electroporation of siRNAs. Cell surface levels of the formyl peptide receptor (FPR1) were measured by binding of the fluorescent ligand FLPEP (see Methods) as assessed by cytometry. Shown are histograms of FLPEP binding (in relative fluorescence units) by cell populations with siRNA targeting FPR1 introduced by electroporation using 0, 0.1, 0.3, or 1.0 μM siRNA.
