# Supplementary material for: Using light to shape chemical gradients for parallel and automated analysis of chemotaxis
Source: Mol Syst Biol. 2015 Apr 23;11(4):804. doi: 10.15252/msb.20156027 (PMC4422560; doi:10.15252/msb.20156027)
Supplement: Supplementary file 3 [file msb0011-0804-sd3.pdf]

Supplementary Figure 3

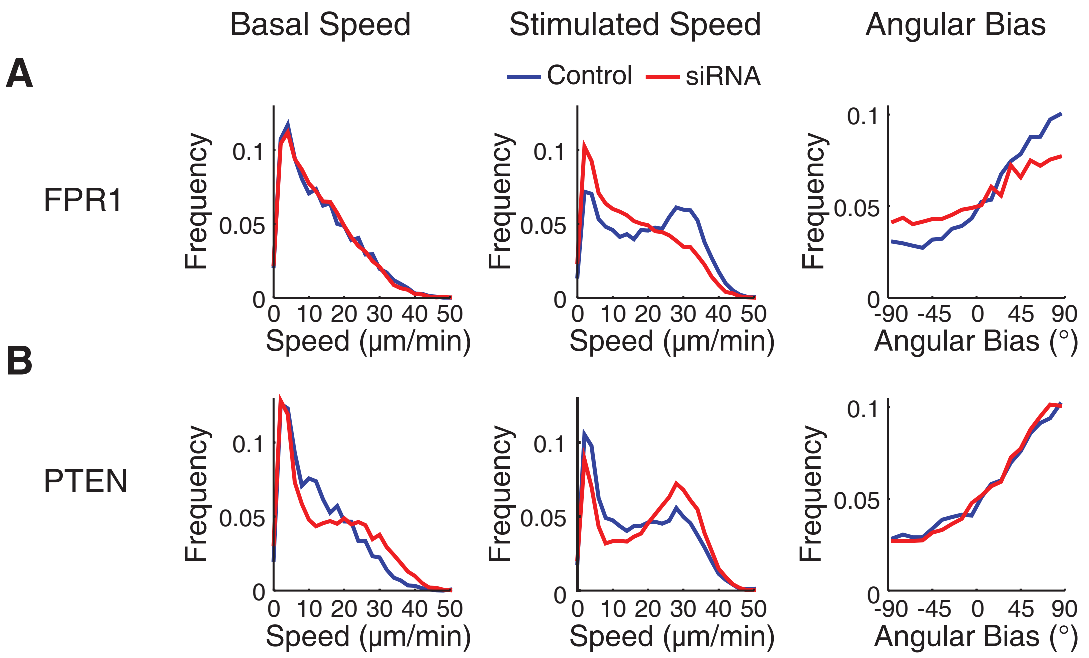

Supplementary Figure S3. Distributions of instantaneous cell movement parameters for individual cells treated with siRNA

A,B, Cell speed and directionality (angular bias) were measured for individual cells for individual frame to frame steps. Shown are histograms of instantaneous cell speed before chemoattractant gradient generation by uncaging of Nv-fMLF (left), cell speed after gradient generation (middle), and angular bias after gradient generation (right). Data cells treated with siRNA targeting FPR1 (A) or PTEN (B) are indicated with red curves, while data for the corresponding in well controls is shown in blue. Each of the plots includes pooled data from three independent single-well experiments.
