# Supplementary material for: Using light to shape chemical gradients for parallel and automated analysis of chemotaxis
Source: Mol Syst Biol. 2015 Apr 23;11(4):804. doi: 10.15252/msb.20156027 (PMC4422560; doi:10.15252/msb.20156027)
Supplement: Supplementary file 4 [file msb0011-0804-sd4.pdf]

Supplementary Figure 4

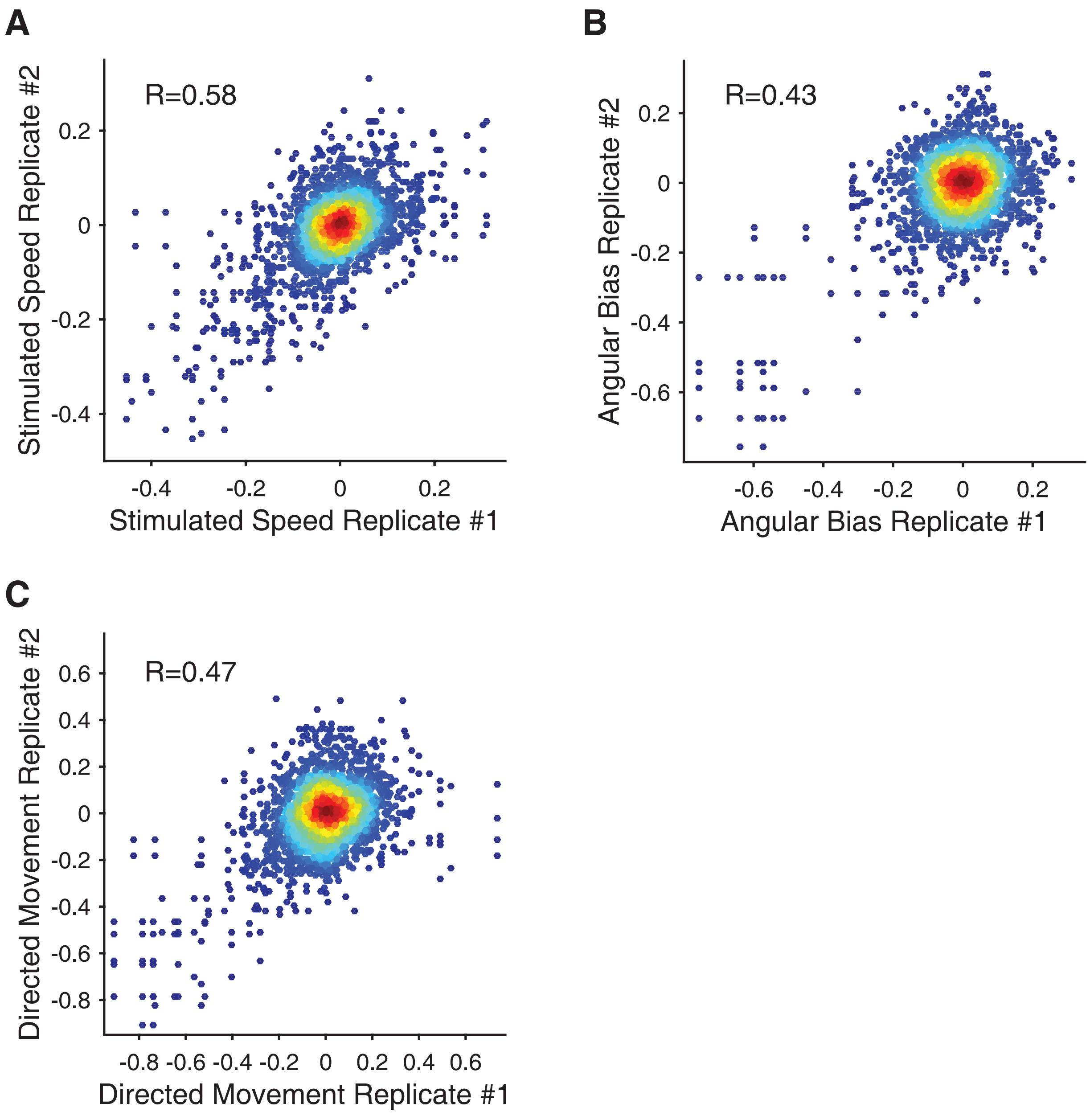

Supplementary Figure S4. Reproducibility of replicate measurements for siRNA phenotypes.
A,B,C, Density-colored scatter plots are shown for measurements from pairs of independent wells with identical siRNA conditions (including data for 285 different siRNA conditions) of normalized phenotypes for stimulated speed (A), angular bias (B), and directed movement (C). In each case, the Pearson's correlation coefficient is indicated on the corresponding graph (0.58, 0.43, and 0.47). See Dataset S1 for a full table of results.
