# Supplementary material for: Using light to shape chemical gradients for parallel and automated analysis of chemotaxis
Source: Mol Syst Biol. 2015 Apr 23;11(4):804. doi: 10.15252/msb.20156027 (PMC4422560; doi:10.15252/msb.20156027)
Supplement: Supplementary file 5 [file msb0011-0804-sd5.pdf]

# Supplementary Figure 5

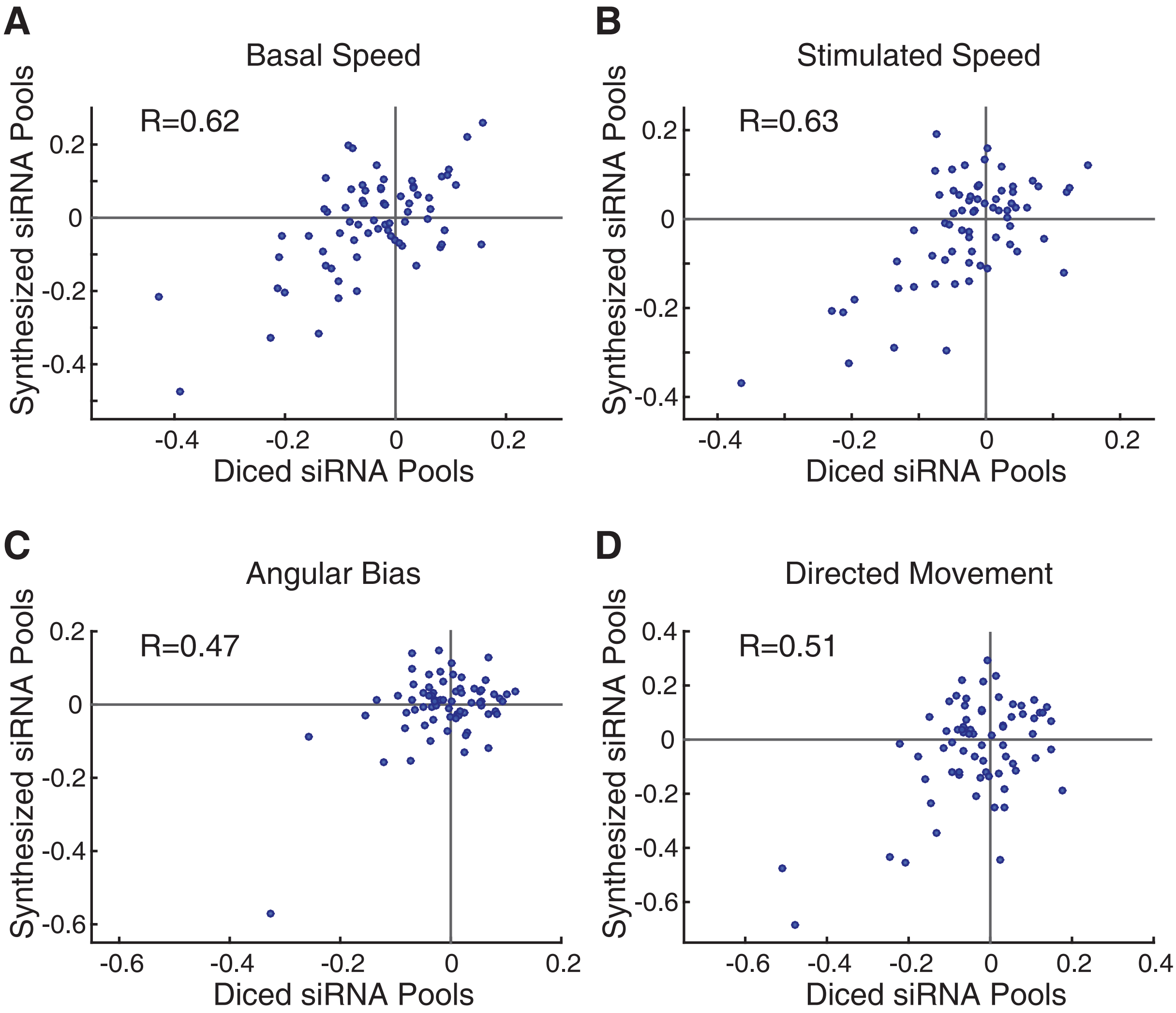


**Supplementary Figure S5. Consistent results for diced and synthesized siRNA pools.**
**A,B,C,D,** Scatter plots for mean normalized phenotypes for diced and synthetic siRNAs pools targeting the same gene. We had both diced and synthetic siRNA pools for 69 different target genes. Plots are shown for basal speed (A), stimulated speed (B), angular bias (C), and directed movement (D). In each case, the Pearson's correlation coefficient is indicated on the corresponding graph (0.62, 0.63, 0.47, and 0.51). See Dataset S2 for a full table of results.
