# Supplementary material for: Using light to shape chemical gradients for parallel and automated analysis of chemotaxis
Source: Mol Syst Biol. 2015 Apr 23;11(4):804. doi: 10.15252/msb.20156027 (PMC4422560; doi:10.15252/msb.20156027)
Supplement: Supplementary file 6 [file msb0011-0804-sd6.pdf]

Supplementary Figure 6

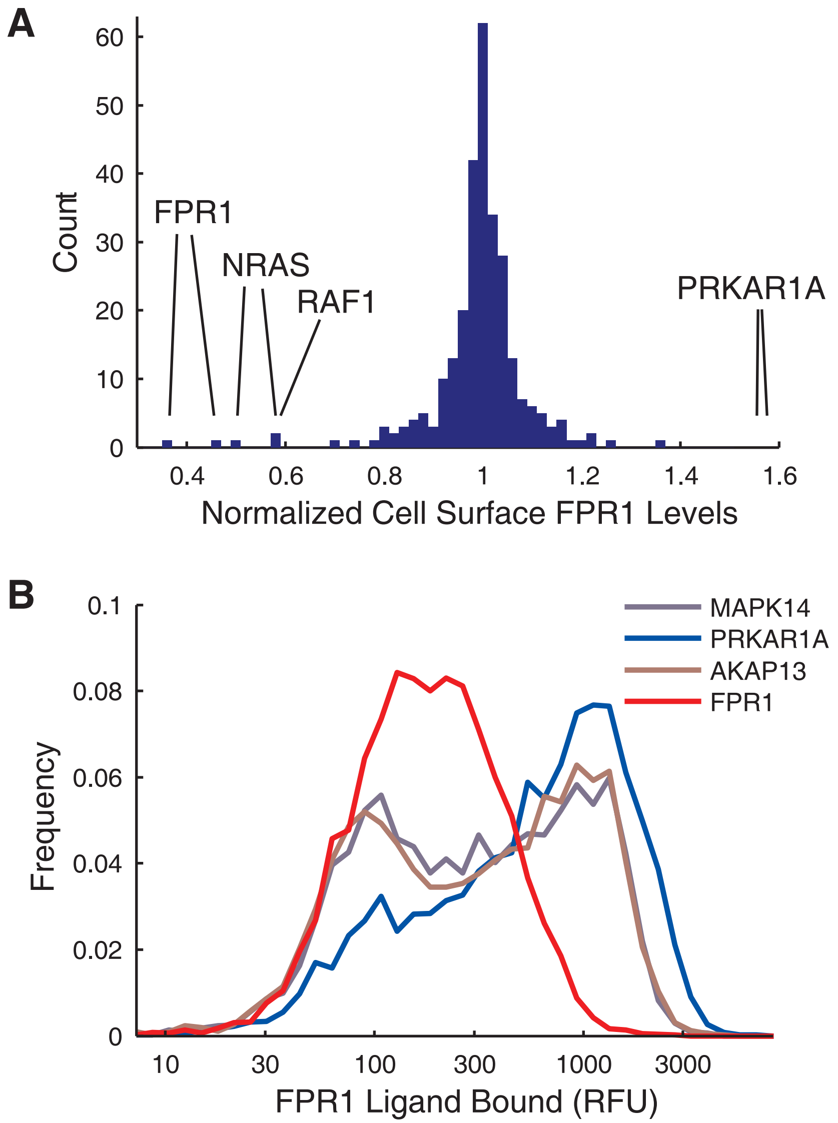

Supplementary Figure S6. Effects of siRNA perturbations on differentiation of PLB-985 cells.
Cell surface levels of the formyl peptide receptor were measured as a marker for differentiation of PLB-985 cells into a neutrophil-like state by measuring binding of the fluorescent FPR1 ligand FLPEP by cytometry (see Methods).
A, Histogram of the normalized mean fluorescent intensities of cell populations treated with each of the 285 siRNAs conditions used in this study. The genes targeted by the siRNAs with the strongest effects are labeled. siRNA targeting FPR1 had the strongest effect, although this is likely through direct targeting of FPR1, rather than an effect on differentiation. A full table of the results is included in Supplementary Dataset S3.
B, Histogram of the distribution of fluorescent intensities for single cells treated with siRNAs targeting FPR1 (red), PRKAR1A (blue), MAPK14 (gray), and AKAP13 (brown). The siRNAs targeting FPR1 and PRKAR1A had the strongest effects on receptor levels (in opposite directions) of any siRNAs used in the study. The siRNAs targeting MAPK14 and AKAP13 were chosen as examples of siRNAs having minimal effects on receptor levels. Ligand binding was measured in relative fluorescence units.
