# Supplementary material for: Using light to shape chemical gradients for parallel and automated analysis of chemotaxis
Source: Mol Syst Biol. 2015 Apr 23;11(4):804. doi: 10.15252/msb.20156027 (PMC4422560; doi:10.15252/msb.20156027)
Supplement: Supplementary file 7 [file msb0011-0804-sd7.pdf]

Supplementary Figure 7

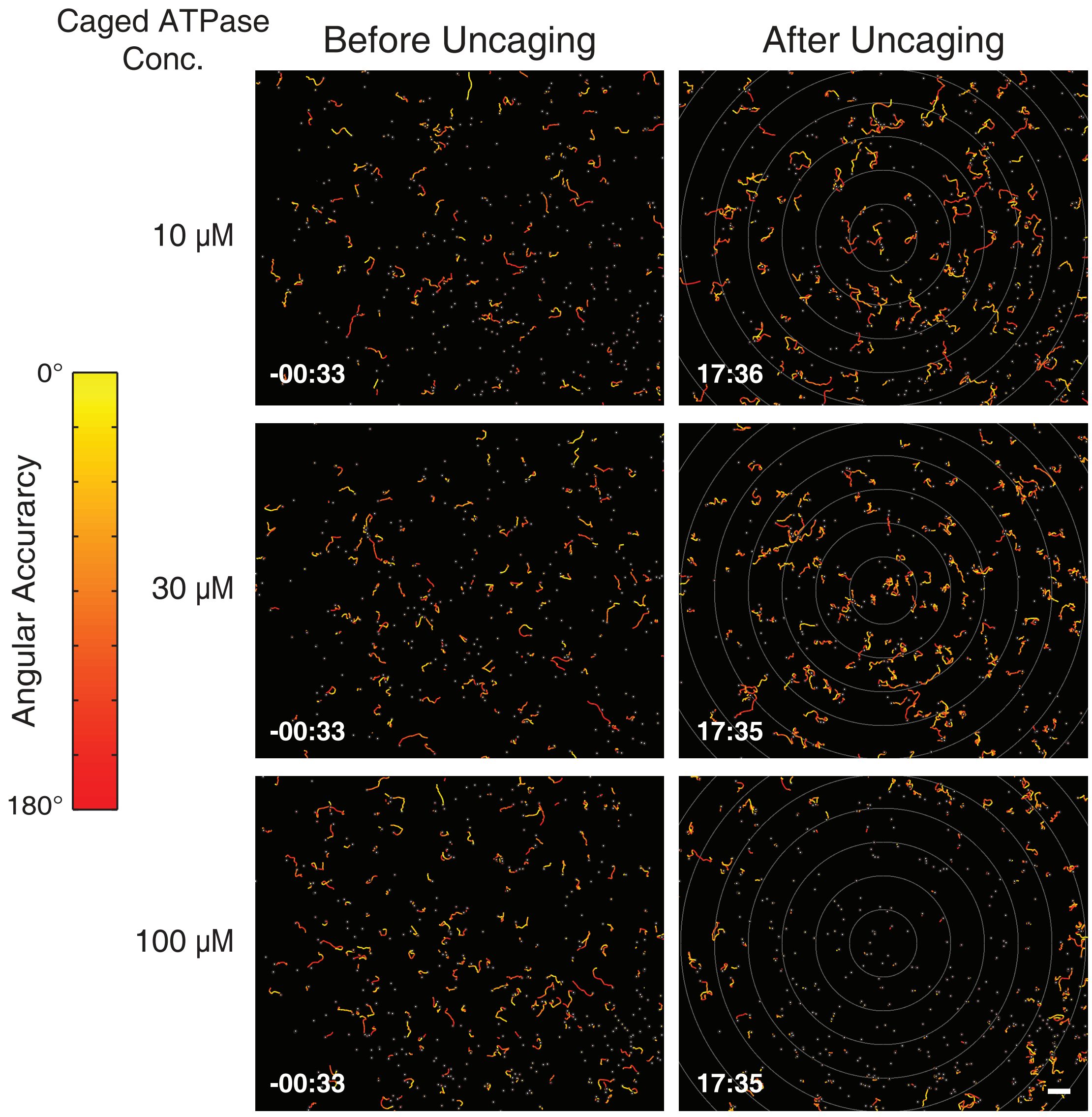

Supplementary Figure S7. Dose-dependent response of differentiated PLB-985 cells to gradients of ATP generated by uncaging. Shown are single images of cell nuclei in the last frame before uncaging (left) and the last frame after uncaging (right). Cell tracks are overlaid and colored according to cell direction towards (light yellow) or away (red) from the gradient center. The scale bar represents a 100 micron length, and the times in the lower left of each image represent the time of the image relative to gradient generation in minutes and seconds. In the right frames, the gradient is indicated by concentric circles representing curves of approximately equal attractant concentration.
