# Supplementary material for: Using light to shape chemical gradients for parallel and automated analysis of chemotaxis
Source: Mol Syst Biol. 2015 Apr 23;11(4):804. doi: 10.15252/msb.20156027 (PMC4422560; doi:10.15252/msb.20156027)
Supplement: Supplementary file 13 [file msb0011-0804-sd13.docx]

**Supplementary Legends**

**Supplementary Figure S1. Dose-dependent chemotactic responses to gradients of fMLF generated by uncaging.**

**A,B,C,** Mean cell speed (A), angular bias (B), and directed speed (C) before (blue) and after (red) generation of gradients from the indicated concentrations of caged fMLF (Nv-fMLF).

**Supplementary Figure S2. Dose-dependent knockdown of FPR1 by electroporation of siRNAs.**

Cell surface levels of the formyl peptide receptor (FPR1) were measured by binding of the fluorescent ligand FLPEP (see Methods) as assessed by cytometry. Shown are histograms of FLPEP binding (in relative fluorescence units) by cell populations with siRNA targeting FPR1 introduced by electroporation using 0, 0.1, 0.3, or 1.0 μM siRNA.

**Supplementary Figure S3. Distributions of instantaneous cell movement parameters for individual cells treated with siRNA**

**A,B,** Cell speed and directionality (angular bias) were measured for individual cells for individual frame to frame steps. Shown are histograms of instantaneous cell speed before chemoattractant gradient generation by uncaging of Nv-fMLF (left), cell speed after gradient generation (middle), and angular bias after gradient generation (right). Data cells treated with siRNA targeting FPR1 (A) or PTEN (B) are indicated with red curves, while data for the corresponding in well controls is shown in blue. Each of the plots includes pooled data from three independent single-well experiments.

**Supplementary Figure S4. Reproducibility of replicate measurements for siRNA phenotypes.**

**A,B,C,** Density-colored scatter plots are shown for measurements from pairs of independent wells with identical siRNA conditions (including data for 285 different siRNA conditions) of normalized phenotypes for stimulated speed (A), angular bias (B), and directed movement (C). In each case, the Pearson’s correlation coefficient is indicated on the corresponding graph (0.58, 0.43, and 0.47). See Supplementary Dataset S1 for a full table of results.

**Supplementary Figure S5. Consistent results for diced and synthesized siRNA pools.**

**A,B,C,D,** Scatter plots for mean normalized phenotypes for diced and synthetic siRNAs pools targeting the same gene. We had both diced and synthetic siRNA pools for 69 different target genes. Plots are shown for basal speed (A), stimulated speed (B), angular bias (C), and directed movement (D). In each case, the Pearson’s correlation coefficient is indicated on the corresponding graph (0.62, 0.63, 0.47, and 0.51). See Supplementary Dataset S2 for a full table of results.

**Supplementary Figure S6. Effects of siRNA perturbations on differentiation of PLB-985 cells.**

Cell surface levels of the formyl peptide receptor were measured as a marker for differentiation of PLB-985 cells into a neutrophil-like state by measuring binding of the fluorescent FPR1 ligand FLPEP by cytometry (see Methods).

**A,** Histogram of the normalized mean fluorescent intensities of cell populations treated with each of the 285 siRNAs conditions used in this study. The genes targeted by the siRNAs with the strongest effects are labeled. siRNA targeting FPR1 had the strongest effect, although this is likely through direct targeting of FPR1, rather than an effect on differentiation. A full table of the results is included in Supplementary Dataset S3.

**B,** Histogram of the distribution of fluorescent intensities for single cells treated with siRNAs targeting FPR1 (red), PRKAR1A (blue), MAPK14 (gray), and AKAP13 (brown). The siRNAs targeting FPR1 and PRKAR1A had the strongest effects on receptor levels (in opposite directions) of any siRNAs used in the study. The siRNAs targeting MAPK14 and AKAP13 were chosen as examples of siRNAs having minimal effects on receptor levels. Ligand binding was measured in relative fluorescence units.

**Supplementary Figure S7. Dose-dependent response of differentiated PLB-985 cells to gradients of ATP generated by uncaging.**

Shown are single images of cell nuclei in the last frame before uncaging (left) and the last frame after uncaging (right). Cell tracks are overlaid and colored according to cell direction towards (light yellow) or away (red) from the gradient center. The scale bar represents a 100 micron length, and the times in the lower left of each image represent the time of the image relative to gradient generation in minutes and seconds. In the right frames, the gradient is indicated by concentric circles representing curves of approximately equal attractant concentration.

**Supplementary Dataset S1. Systematic Chemotaxis Data for siRNA Perturbations - Independent Replicates**

This dataset contains quantitative measurements of each chemotaxis movement parameter for each of the 285 distinct siRNA conditions used in this study in each independent replicate measurement. Each row corresponds to one siRNA perturbation, and measurements from different independent experiments are given in separate columns.

**Supplementary Dataset S2. Systematic Chemotaxis Data for siRNA Perturbations - Final Results**

This dataset contains the final (averaged) quantitative measurements of each chemotaxis movement parameter for each of the 285 distinct siRNA conditions used in this study. Each row corresponds to one siRNA perturbation. This data table was obtained by averaging (taking the mean) the data in Supplementary Dataset S1.

**Supplementary Dataset S3. Systematic Differentiation Data for siRNA Perturbations**

Cell surface levels of the formyl peptide receptor were measured as a marker for differentiation of PLB-985 cells into a neutrophil-like state by measuring binding of the fluorescent FPR1 ligand FLPEP by cytometry (see Methods). This dataset contains the normalized FLPEP binding for each of the 285 distinct siRNA conditions used in this study. The mean, standard deviation, and all measurements from independent experiments are included in the table. Each row corresponds to one siRNA condition. This data corresponds to that shown in Supplementary Fig S6.

**Supplementary Movie S1. Chemotaxis assay with Nv-fMLF**

This movie shows time lapse images of differentiated PLB-985 cells expressing an mCherry-Histone H2B fusion protein responding to a gradient of fMLF induced by uncaging of Nv-fMLF. The time relative to gradient induction is shown in the upper right corner. When the gradient is present, concentric green circles indicate curves of approximately equal fMLF concentration. Tracks of cell movement for the preceding 6 frame-to-frame steps are shown in orange.

**Supplementary Movie S2. Chemotaxis assay with NPE-ATP**

Shown are three movies (side-by-side) analogous to that in Supplementary Movie S1, but here of cells responding to a gradient of ATP generated by uncaging of NPE-ATP. The concentrations of NPE-ATP in the upper left corner of each movie. The cells respond with transient chemotaxis to the gradient with 10 or 30 μM NPE-ATP, but respond by stopping with 100 μM ATP.
