# Supplementary material for: Using light to shape chemical gradients for parallel and automated analysis of chemotaxis
Source: Mol Syst Biol. 2015 Apr 23;11(4):804. doi: 10.15252/msb.20156027 (PMC4422560; doi:10.15252/msb.20156027)
Supplement: Supplementary file 14 [file msb0011-0804-sd14.zip › HT_Chemotaxis_Toolbox-master/ann_source/ANNmanual_1.1.pdf]

# ANN Programming Manual

David M. Mount*
Department of Computer Science and
Institute for Advanced Computer Studies
University of Maryland, College Park, Maryland.

ANN, Version 1.1
Copyright, 2010 David M. Mount

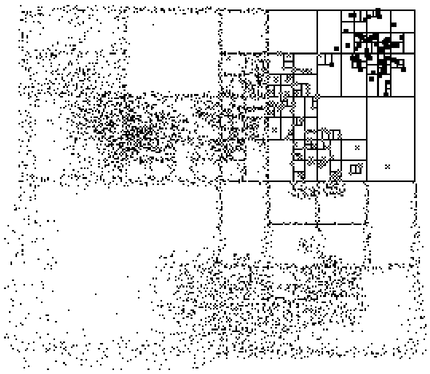

*Email: mount@cs.umd.edu. The support of the National Science Foundation under grants CCR–9712379 and CCR-0098151 is gratefully acknowledged.

# Contents


**1 Introduction** **1**
- 1.1 What is ANN? . . . . . 1
- 1.2 Downloading and Using ANN . . . . . 1
- 1.3 Compiling ANN . . . . . 2
  - 1.3.1 Compiling on Unix/Linux Systems . . . . . 2
  - 1.3.2 Compiling on Microsoft Windows Systems . . . . . 2
  - 1.3.3 Precompiled Files for Microsoft Windows . . . . . 3
- 1.4 Compiling Applications that Use the Library . . . . . 3

**2 The ANN Library** **4**
- 2.1 Using ANN . . . . . 5
  - 2.1.1 Coordinates, Points, Distances, and Indices . . . . . 5
  - 2.1.2 Nearest Neighbor Search Structure . . . . . 6
  - 2.1.3 Point Utility Procedures . . . . . 9
  - 2.1.4 A Sample Program . . . . . 10
  - 2.1.5 Compiling Sample Applications that use ANN . . . . . 11
- 2.2 Configuring ANN . . . . . 13
  - 2.2.1 Norms and Distances . . . . . 14
  - 2.2.2 More on Points, Cordinates, and Distances . . . . . 15
  - 2.2.3 Self Matches . . . . . 16
- 2.3 Modifying the Search Structure . . . . . 16
  - 2.3.1 ANN Data Structures . . . . . 16
  - 2.3.2 Internal Tree Structure . . . . . 18
  - 2.3.3 Splitting Rules for kd-trees . . . . . 18
  - 2.3.4 Shrinking Rules for bd-trees . . . . . 20
- 2.4 Search Algorithms . . . . . 21
  - 2.4.1 Standard Search . . . . . 21
  - 2.4.2 Priority Search . . . . . 22
  - 2.4.3 Early Termination Option . . . . . 22
- 2.5 Search Tree Utility Functions . . . . . 23
  - 2.5.1 Printing the Search Structure . . . . . 23
  - 2.5.2 Saving and Restoring the Search Structure on a File . . . . . 23
  - 2.5.3 Gathering Statistics on Tree Structure . . . . . 25
- 2.6 Compile-Time Options . . . . . 25

**3 Utility Programs** **27**
- 3.1 ann_test: A Test and Evaluation Program . . . . . 27
  - 3.1.1 Operations . . . . . 27
  - 3.1.2 Options Affecting Tree Structure: . . . . . 28
  - 3.1.3 Options Affecting Data and Query Point Generation . . . . . 29
  - 3.1.4 Options Affecting Nearest Neighbor Searching . . . . . 31
  - 3.1.5 Options Affecting the Amount of Output . . . . . 31
  - 3.1.6 Options Affecting the Validation . . . . . 32
- 3.2 ann2fig: Visualization Tool . . . . . 33

# 1 Introduction

## 1.1 What is ANN?

ANN is a library written in the C++ programming language to support both exact and approximate nearest neighbor searching in spaces of various dimensions. It was implemented by David M. Mount of the University of Maryland and Sunil Arya of the Hong Kong University of Science and Technology. ANN (pronounced like the name "Ann") stands for the *Approximate Nearest Neighbor* library. ANN is also a testbed containing programs and procedures for generating data sets, collecting and analyzing statistics on the performance of nearest neighbor algorithms and data structures, and visualizing the geometric structure of these data structures.

In the *nearest neighbor problem* a set $P$ of data points in $d$-dimensional space is given. These points are preprocessed into a data structure, so that given any query point $q$, the nearest (or generally $k$ nearest) points of $P$ to $q$ can be reported efficiently. ANN is designed for data sets that are small enough that the search structure can be stored in main memory (in contrast to approaches from databases that assume that the data resides in secondary storage). Points are assumed to be represented as coordinate vectors of reals (or integers). The distance between two points can be defined in many ways. ANN assumes that distances are measured using any class of distance functions called *Minkowski metrics*. These include the well known Euclidean distance, Manhattan distance, and max distance.

Answering nearest neighbor queries efficiently, especially in higher dimensions, seems to be very difficult problem. It is always possible to answer any query by a simple brute-force process of computing the distances between the query point and each of the data points, but this may be too slow for many applications that require that a large number of queries be answered on the same data set. Instead the approach is to preprocess a set of data points into a data structure from which nearest neighbor queries are then answered. There are a number of data structures that have been proposed for solving this problem. See, for example, [AMN$^+$98, Ben90, Cla97, Kle97, PS85, Spr91], for more information on this problem.

One difficulty with exact nearest neighbor searching is that for virtually all methods other than brute-force search, the running time or space grows exponentially as a function of dimension. Consequently these methods are often not significantly better than brute-force search, except in fairly small dimensions. However, it has been shown by Arya and Mount [AM93b] and Arya, et al. [AMN$^+$98] that if the user is willing to tolerate a small amount of error in the search (returning a point that may not be the nearest neighbor, but is not significantly further away from the query point than the true nearest neighbor) then it is possible to achieve significant improvements in running time. ANN is a system for answering nearest neighbor queries both *exactly* and *approximately*.

This manual describes how to download and install ANN, how to use the library, how to change its configuration for different distance functions and data representations, and finally how to use its utility programs for testing and visualizing the data structure.

## 1.2 Downloading and Using ANN

The current version of ANN is version 1.1. The ANN source code and documentation is available from the ANN web page:

`http://www.cs.umd.edu/~mount/ANN`

The unbundled software consists of the following major files and directories.

ReadMe.txt: General description of the library.

Copyright.txt: Copyright information.

License.txt: Conditions of use for the library.

include: Include files for compiling programs that use the library.

src: The source files for the library.

sample: A small sample program, which illustrates how to input a point set, build a search structure for the set, and answer nearest neighbor queries. (See Section 2.1.4.)

test: A program that provides a simple script input for building search trees and comparing their performance on point sets that are either read from an input file or generated according to some probability distribution. (See Section 3.1.)

ann2fig: A program that generates a visual representation (in fig format) of the tree's spatial decomposition. (See Section 3.2.)

lib: Where the compiled library is stored.

bin: Where the compiled executables are stored.

doc: This documentation.

MS_Win32: Solution and project files for compilation under Microsoft Visual Studio .NET.

## 1.3 Compiling ANN

ANN requires an ANSI standard C++ compiler. It has been compiled successfully on Unix and Linux systems including Sun Workstations running SunOS 5.X (Solaris), Linux Red Hat 2.X, and on DEC Alphas running Digital Unix v4.X, on SGIs running IRIX 6.X. Makefiles for all these systems. It has also been compiled under Microsoft Windows XP (under Visual Studio .NET).

### 1.3.1 Compiling on Unix/Linux Systems

After downloading the sources, change to the ANN root directory (from which directories such as bin, doc, and include branch off) and enter the command "make". This will provide a list of platforms and options under which to compile ANN. If you do not see your particular configuration on this list, then you might try modifying the file Make-config for your particular system. The authors welcome any additions to the list of supported platforms.

There are some additional compilation options that can be enabled or disabled. These are described in Section 2.6. To recompile the library, enter the command "make realclean" to delete the library and utility programs, and then reenter the make command for compilation.

### 1.3.2 Compiling on Microsoft Windows Systems

To compile under Visual C++ running within Microsoft Visual Studio .NET, go to the directory MS_Win32. Open the solution file Ann.sln, select the "Release" configuration, and select "Build → Build Solution." After compilation, the file ANN.lib is stored in the directory MS_Win32\dll\Release. The file ANN.dll file and executables of the utility programs are stored in the directory bin (relative to the ANN root directory). These two files, along with the files in the directory include/ANN are needed for compiling applications that use ANN.

### 1.3.3 Precompiled Files for Microsoft Windows

For Microsoft Windows users that do not need to modify the software, a bundle containing all the files you need to use ANN has been provided. This contains the include files in include/ANN along with ANN.lib and ANN.dll. It can be downloaded directly from http://www.cs.umd.edu/~mount/ANN. In order to use these with an application it is necessary to copy each of these files to appropriate directories for the compiler and linker to locate them, and then to set the appropriate compiler, linker, and path settings so the system can locate all these files. You should consult your local system documentation for this information. An example of how to do this for the sample program is given in Section 2.1.5.

## 1.4 Compiling Applications that Use the Library

In order to use ANN in a C++ program, the program must include the header file for ANN, which contains the declarations for the ANN objects and procedures. This is done with the following statement in the C++ source code.

```
#include <ANN/ANN.h>
```

This assumes that the ANN include directory is already on the compiler's search path for include files. On most Unix/Linux-based C++ compilers, this is done by setting the "-I" (capital letter "i") option on the compilation command line to point to the include directory in the ANN base directory.

Then the program is linked with the ANN library. On Unix/Linux-based systems this is done with the "-l" (lower-case letter "$\ell$") option, assuming that the library search path has been set to include the ANN library directory. The library search path can be set with the "-L" option. For example, on my Unix system, the following command line could be used to compile the program my_program.cpp using the ANN library and the GNU C++ compiler. Let ann denote the path to root ANN directory.

```
g++ my_program.cpp -Iann/include -Lann/lib -lANN
```

Some additional information on compiling applications that use ANN on Microsoft Windows systems can be found in Section 2.1.5.

## 2 The ANN Library

ANN is a library of C++ objects and procedures that supports approximate nearest neighbor searching. In *nearest neighbor searching*, we are given a set of data points $S$ in real $d$-dimensional space, $\mathbb{R}^d$, and are to build a data structure such that, given any query point $q \in \mathbb{R}^d$, the nearest data point to $q$ can be found efficiently. In general, we are given $k \geq 1$, and are asked to return the $k$-nearest neighbors to $q$ in $S$.

In *approximate nearest neighbor searching*, an error bound $\epsilon \geq 0$ is also given. The search algorithm returns $k$ distinct points of $S$, such that for $1 \leq i \leq k$, the ratio between the distance to the $i$th reported point and the true $i$th nearest neighbor is at most $1 + \epsilon$.

ANN's features include the following.

- It supports $k$-nearest neighbor searching, by specifying $k$ with the query.
- It supports both exact and approximate nearest neighbor searching, by specifying an approximation factor $\epsilon \geq 0$ with the query.
- It supports any Minkowski distance metric, including the $L_1$ (Manhattan), $L_2$ (Euclidean), and $L_\infty$ (Max) metrics.
- Preprocessing time and space are both linear in the number of points $n$ and the dimension $d$, and are independent of $\epsilon$. Thus the data structure requires storage that is only moderately larger than the underlying data set.

ANN was written as a testbed for a class of nearest neighbor searching algorithms, particularly those based on orthogonal decompositions of space. These include kd-trees [Ben90, FBF77], and box-decomposition trees [AMN$^+$98]. These will be described in Section 2.3.1. The library supports a number of different methods for building these search structures. It also supports two methods for searching these structures: standard tree-ordered search [AM93a] and priority search [AMN$^+$98]. These will be described in Section 2.4.

In addition to the library there are two programs provided for testing and evaluating the performance of various search methods. The first, called ann_test, provides a primitive script language that allows the user to generate data sets and query sets, either by reading from a file or randomly through the use of a number of built-in point distributions. Any of a number of nearest neighbor search structures can be built, and queries can be run through this structure. This program outputs a number of performance statistics, including the average execution time, number of nodes visited in the data structure, and the average error made in the case of approximate nearest neighbors. An operation is provided to have the data structure dumped to a file and to load the data structure from a dumped file.

The second program, called ann2fig, takes the dumped data structure and generates an illustration of the data structure, which is output to a file in a simple graphics format. When the dimension is higher than two, this program can output any planar 2-dimensional "slice" of the data structure. An example is shown in Figure 1. The output of ann2fig is the same format used by the Unix program xfig. Through the Unix program fig2dev, it is possible to convert this format to a number of other picture formats, including encapsulated postscript.

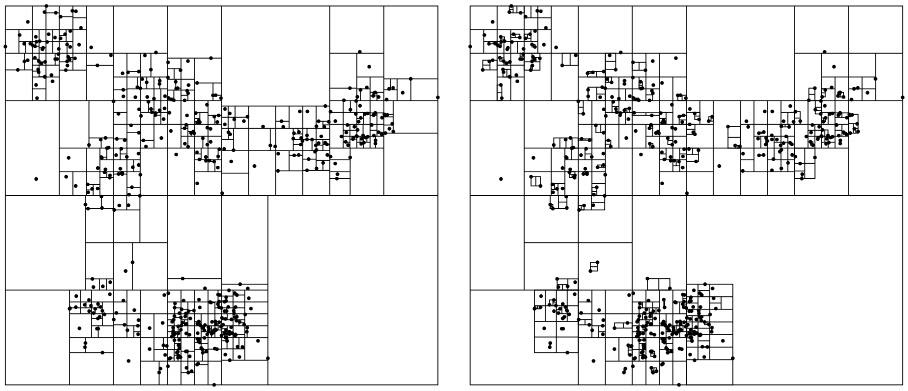

Figure 1: Sample output of ann2fig for a kd-tree and a box-decomposition tree.

## 2.1 Using ANN

This section discusses how to use ANN for answering nearest neighbors in its default configuration, namely computing the nearest neighbor using Euclidean distance for points whose coordinates are of type double. Later in Section 2.2 we discuss how to customize ANN for different coordinate types and different norms.

### 2.1.1 Coordinates, Points, Distances, and Indices

Each point in $d$-dimensional space is assumed to be expressed as a $d$-vector of coordinates

$$p = (p_0, p_1, \ldots, p_{d-1}).$$

(Indices start from 0, as is C++'s convention.) A coordinate is of type ANNcoord. By default, ANNcoord is defined to be of type double, but it is possible to modify the ANN include files to change this and other ANN types, as described in Section 2.2.2.

ANN defines the distance between two points to be their Euclidean distance, that is

$$\text{dist}(p, q) = \left( \sum_{0 \le i < d} (p_i - q_i)^2 \right)^{1/2}.$$

(Section 2.2.1 explains how to modify ANN for other Minkowski norms.) For the purposes of comparing distances, it is not necessary to compute the final square root in the above expression. ANN actually computes *squared distances* rather than true Euclidean distances. A squared distance is defined to be of type ANNdist. It is defined to be double by default (but can be changed). By using squared distances rather than true Euclidean distances, ANN not only saves on the time of computing square roots, but has the advantage that integer types can be used instead to accurately represent distances when integer type coordinates are used. (It worth emphasizing that, even though ANN represents distances internally as squared distances, when it computes $\epsilon$-approximate nearest neighbors, the approximation is relative to the true, not squared, distance.)

The most basic object manipulated by ANN is a *point*, which is defined in C++ to be a dimensionless array of coordinates, or more simply a pointer to a coordinate.[1] Thus we define ANNpoint to be

[1]It is natural to wonder why ANN does not define a special point class, which might store additional information

```
typedef ANNcoord* ANNpoint;          // a point
```

It is the user's responsibility to allocate and deallocate storage for points. Each point must have at least as many components allocated as the dimension of the space. Any extra components are ignored.

Since ANN operates on arrays of points and distances, we also define dimensionless arrays of these two objects:

```
typedef ANNpoint* ANNpointArray;    // an array of points
typedef ANNdist*  ANNdistArray;     // an array of squared distances
```

We will see later that a set of data points is presented to ANN as an ANNpointArray. ANN returns the results of a nearest neighbor query as an integer index into this array. To make it clear when we are doing so, ANN defines the type ANNidx to be a psuedonym for int. The result of a $k$-nearest neighbor query is returned as a pointer to an array of indices, which is of type ANNidxArray, defined as follows.

```
typedef ANNidx*   ANNidxArray;      // an array of point indices
```

Finally, ANN provides a boolean type called ANNbool. (Although ANSI C++ provides a type bool, this is not supported in some of the older compilers.) Its values are ANNfalse and ANNtrue.

### 2.1.2 Nearest Neighbor Search Structure

The principal structure that ANN uses for performing nearest neighbor searches is called an ANNpointSet. This is an abstract object which supports two search operations, annkSearch(), which computes the approximate $k$ nearest neighbors of a query point, and annKFRSearch(), which performs a combination of a fixed-radius approximate $k$ nearest neighbor search and a range search (see the description below).

ANN provides three concrete instantiations of an ANNpointSet: ANNbruteForce, ANNkd_tree, and ANNbd_tree. The first of these stores the points as an array, and implements a simple brute-force search for nearest neighbors. It is provided primarily for the use of the implementors to validate the correctness of the more sophisticated search structures, and should not be used for actual nearest neighbor queries. Among the other two, we will first introduce only the more basic one, the ANNkd_tree, and leave the ANNbd_tree to be discussed in Section 2.3. The ANNkd_tree supports the following operations.

**Constructor:** This builds a kd-tree from a set of $n$ data points in dimension $d$, stored in a point array $pa$. The procedure allocates the necessary storage for the data structure. It is assumed that there is at least one data point ($n \geq 1$) and that the dimension is strictly positive ($d \geq 1$). Warning: This procedure does virtually no error checking, and if these assumptions are violated or if storage cannot be allocated, then the most likely result will be that the program aborts.

A (simplified) prototype is given below. (See Section 2.3 for more complete information.) The order in which the points appear in the points array is significant, because nearest neighbors

about points. This would then necessitate that the library be templated about the point type. Since compiler support for templating was rather weak in the mid-90's when ANN was first implemented designed, it was decided instead to decouple the user's point object from the ANN point type. In this way we allow the user to define a point object in any way desired. The user then interfaces with ANN by simply providing a pointer to the point's coordinates. In many cases, this may just be a field in the user's own point class.

are returned by their index in this array. Following C++ conventions, the items are indexed from 0 to $n-1$.

```
ANNkd_tree::ANNkd_tree(
    ANNpointArray   pa,             // data point array
    int             n,              // number of points
    int             d);             // dimension
```

To conserve space, the tree does not actually store the data points, but rather stores pointers to the array *pa*. For this reason the contents of *pa* should be unchanged throughout the lifetime of the data structure.

**$k$-Nearest Neighbor Search:** This member function is given a query point $q$, a nonnegative integer $k$, an array of point indices, *nn_idx*, and an array of distances, *dists*. Both arrays are assumed to contain at least $k$ elements. The procedure computes the $k$ nearest neighbors to $q$ in the point set, and stores the indices of the nearest neighbors (relative to the point array *pa* given in the constructor). The nearest neighbor is stored in *nn_idx*[0], the second nearest in *nn_idx*[1], and so on. The *squared* distances to the corresponding points are stored in the array *dists*.

Optionally, a real value $\epsilon \geq 0$ may be supplied. If so, then the $i$th nearest neighbor is a $(1+\epsilon)$ approximation to the true $i$th nearest neighbor. That is, the true (not squared) distance to this point may exceed the true distance to the real $i$th nearest neighbor of $q$ by a factor of $(1+\epsilon)$. If $\epsilon$ is omitted then nearest neighbors are computed exactly.

ANN supports two different methods for searching the kd-tree. Here we present the simpler one, which is the more efficient for small values of $\epsilon$. Another searching algorithm, called priority search, is presented later in Section 2.4.2.

```
virtual void ANNkd_tree::annkSearch(
    ANNpoint        q,            // query point
    int             k,            // number of near neighbors to find
    ANNidxArray     nn_idx,       // nearest neighbor array (modified)
    ANNdistArray    dists,        // dist to near neighbors (modified)
    double          eps=0.0);     // error bound
```

**Fixed-radius $k$-Nearest Neighbor Search:** This member function is a modification of the above procedure, which searches for up to $k$ nearest neighbors, but confines the search to a fixed radius bound. It is given a query point $q$, a (squared) radius bound *sqRad*, a nonnegative integer $k$. Optionally, it is given an array of point indices, *nn_idx*, an array of distances, *dists*. If provided, both arrays are assumed to contain at least $k$ elements.

This procedure performs two different types of search. First, if $k$ is positive and the two arrays *nn_idx* and *dists* are provided, then among the points whose squared distance from $q$ is at most *sqRad*, it finds the $k$ closest of these to $q$. If the number of points within the squared radius bound is some value $k' < k$, then only the first $k'$ entries of these arrays have meaningful entries. The other entries of *nn_idx* contain the special value *ANN_NULL_IDX* and the other entries of *dists* contain the special value *ANN_DIST_INF* (which are defined in ANN.h).

This is called a fixed-radius search, because it ignores all the points that lie outside of the radius bound. It is not however, a true fixed-radius search, because it computes only the closest $k$ points that lie within the radius bound. Thus, if the value of $k$ is less than the total number of points $k'$ in the radius bound, the farthest $k' - k$ points within the radius will not be reported. (This limitation is because ANN allocates all its arrays statically.)

It is easy, however, to determine whether any points were missed. The procedure returns a count of the total number of points that lie within the radius bound. (This feature is always enabled, even if $k = 0$.) In order to produce a true fixed-radius search, first set $k = 0$ and run the procedure in order to obtain the number $k'$ of points that lie within the radius bound. Then, allocate index and distance arrays of size $k'$ each, and repeat the fixed-radius search by setting $k = k'$ and passing in these two arrays.

Optionally, a real value $\epsilon \geq 0$ may be supplied. If so, the squared radius bound is treated as an approximate quantity in the following sense. Assuming that we are using Euclidean distances, let $r = \sqrt{sqRad}$ be the true radius bound. Every data point whose distance from $q$ is less than $r/(1+\epsilon)$ will be considered, and any point whose distance from $q$ is greater than $r$ will not be considered. The remaining points either may or may not be considered (at the discretion of the search algorithm). Among the points that are considered, the $i$th elements of *nn_idx* and *dists* contain the $i$th closest point to $q$, and the procedure returns a count of all the points under consideration.

Here is the prototype of the fixed-radius search procedure.

```
virtual int ANNkd_tree::annkFRSearch(
    ANNpoint        q,              // query point
    ANNdist         sqRad,          // squared radius
    int             k = 0,          // number of near neighbors to return
    ANNidxArray     nn_idx = NULL,  // nearest neighbor array (modified)
    ANNdistArray    dd = NULL,      // dist to near neighbors (modified)
double              eps = 0.0);     // error bound
```

Unlike annkSearch(), there is no priority search version of of annkFRSearch(). Because it visits all the points in the search radius one by one, the search procedure is rather inefficient if the number of points in the radius bound is large.

**Other Information:** There are three functions provided for extracting information from a search structure. These are particularly useful if the structure has been loaded from a file (through the load constructor). The function theDim() returns the dimension of the space, nPoints() returns the number of points in the structure, and thePoints() returns a pointer to the array of data points.

```
virtual int ANNkd_tree::theDim();   // return the dimension of space
virtual int ANNkd_tree::nPoints();  // return the number of points
                                    // return a pointer to points
virtual ANNpointArray ANNkd_tree::thePoints();
```

**Destructor:** The destructor deallocates the search structure. (It does not deallocate the points.)

```
ANNkd_tree::~ANNkd_tree();
```

**Closing ANN:** The library allocates a small amount of storage, which is shared by all search structures built during the program's lifetime. Because the data is shared, it is not deallocated, even when the all the individual structures are deleted. To avoid the resulting (minor) memory leak, the following function can be called after all search structures have been destroyed. (It is not a member function of the structure.)

```
void annClose();
```

### 2.1.3 Point Utility Procedures

As mentioned earlier ANNpoint, is of type ANNcoord*, a pointer to a dimensionless array of coordinates (see Section 2.1.1). An ANNpointArray is a dimensionless array of such points, that is, of type ANNpoint*. Since a point type does not record its own dimension, all procedures that operate on points must be supplied the dimension. ANN provides a few utility procedures to aid in performing some useful operations on points.

**annDist:** This returns the squared distance between two points $p$ and $q$ in dimension $d$. For reasons of efficiency, ANN does not use this procedure for most of its distance computations.

```
ANNdist annDist(
    int             dim,            // dimension of space
    ANNpoint        p,
    ANNpoint        q);
```

**annAllocPt:** This allocates storage for a point $p$ in dimension $d$. That is, it allocates an array of size $d$ whose elements are of type ANNcoord. In addition to the dimension, it can also be passed a coordinate value (0 by default), which it uses to initialize all coordinates of the point. This procedure returns a pointer to the allocated point, and NULL if storage cannot be allocated for any reason.

```
ANNpoint annAllocPt(
    int             dim,            // dimension of the space
    ANNcoord        c = 0);         // initial coordinate value
```

**annDeallocPt:** Deallocates storage for a point $p$ as allocated by annAllocPt. As a side effect (for safety) it assigns $p$ the value NULL.

```
void annDeallocPt(
    ANNpoint&        p);            // (set to NULL on return)
```

**annAllocPts:** This is used to allocate an array of points. It first allocates an array of $n$ pointers to points (each element is of type ANNpoint), and for each element it allocates storage for $d$ coordinates, and stores a pointer to this storage in the element. It returns a pointer to this array of pointers. It performs no initialization of the coordinate values.

```
ANNpointArray annAllocPts(
    int             n,              // number of points
    int             dim);           // dimension
```

**annDeallocPts:** This deallocates the storage allocated by annAllocPts. This procedure should only be applied to arrays of points allocated by annAllocPts. As a side effect (for safety) it assigns $pa$ the value NULL.

```
void annDeallocPts(
    ANNpointArray&   pa);           // (set to NULL on return)
```

**annCopyPt:** This makes a copy of a point by first allocating storage for a new point and then copying the contents of the source point to the new point. A pointer to the newly allocated point is returned.

```
ANNpoint annCopyPt(
    int             dim,            // dimension
    ANNpoint        source);        // point to copy
```

### 2.1.4 A Sample Program

In this section we present is a sample program demonstrating the basic elements of ANN. The program inputs command line arguments such as the dimension $d$, the number of nearest neighbors $k$, the error bound $\epsilon$, and the file names containing the query and data point. The program allocates storage for data points, one query point, and results, consisting of the nearest neighbor indices and the distances. The program inputs the data points and builds a kd-tree search structure for these points. Then it reads query points, and for each computes $k$ approximate nearest neighbors with error bound $\epsilon$, and outputs the results.

The presentation below shows only the most relevant elements of the program. The complete source can be found in sample/ann_sample.cpp. (All file names are given relative to the ANN root directory.) To simplify the presentation, the procedures getArgs() and readPt() have been omitted. The first reads command line arguments and initializes the global parameters. The second reads a single point from an input stream, and returns false if the end of file is encountered.

```
#include <cstdlib>                      // C standard library
#include <fstream>                      // file I/O
#include <ANN/ANN.h>                    // ANN declarations

using namespace std;                    // make std:: accessible

void getArgs(int argc, char **argv) { ... }     // get command-line arguments
bool readPt(istream& in, ANNpoint p) { ... }    // read point (false on EOF)

//
// Global variables, initialized in getArgs
//
int             k               = 1;            // number of nearest neighbors
int             dim             = 2;            // dimension
double          eps             = 0;            // error bound
int             maxPts          = 1000;         // maximum number of data points
istream*        dataIn          = NULL;         // input for data points
istream*        queryIn         = NULL;         // input for query points

int main(int argc, char **argv)
{
    int                 nPts;                   // actual number of data points
    ANNpointArray       dataPts;                // data points
    ANNpoint            queryPt;                // query point
```

```
    ANNidxArray         nnIdx;                  // near neighbor indices
    ANNdistArray        dists;                  // near neighbor distances
    ANNkd_tree*         kdTree;                 // search structure

    getArgs(argc, argv);                        // read command-line arguments

    queryPt = annAllocPt(dim);                  // allocate query point
    dataPts = annAllocPts(maxPts, dim);         // allocate data points
    nnIdx = new ANNidx[k];                      // allocate near neigh indices
    dists = new ANNdist[k];                     // allocate near neighbor dists

    nPts = 0;                                   // read data points
    while (nPts < maxPts && readPt(*dataIn, dataPts[nPts])) nPts++;

    kdTree = new ANNkd_tree(                    // build search structure
                    dataPts,                    // the data points
                    nPts,                       // number of points
                    dim);                       // dimension of space

    while (readPt(*queryIn, queryPt)) {         // read query points

        kdTree->annkSearch(                     // search
                queryPt,                        // query point
                k,                              // number of near neighbors
                nnIdx,                          // nearest neighbors (returned)
                dists,                          // distance (returned)
                eps);                           // error bound

        cout << "NN:  Index  Distance\n";
        for (int i = 0; i < k; i++) {           // print summary
            dists[i] = sqrt(dists[i]);          // unsquare distance
            cout << i << "  " << nnIdx[i] << "  " << dists[i] << "\n";
        }
    }
    delete [] nnIdx;                            // clean things up
    delete [] dists;
    delete kdTree;
    annClose();                                 // done with ANN

    return EXIT_SUCCESS;
}
```

### 2.1.5 Compiling Sample Applications that use ANN

The sample program is typical in structure to applications that use ANN. If you are on a Unix/Linux system, the sample program is automatically compiled when you the file sample/Makefile can be used to compile the program (perhaps with minor modifications needed, depending on your system). Assuming that ANN has already been compiled, the GNU g++ compiler is used, and ann denotes the path to root ANN directory, the sample program can be compiled using:

```
g++ ann_sample.cpp -o ann_sample -Iann/include -Lann/lib -lANN
```

If you are working on a typical Microsoft Windows system with Microsoft Visual Studio .NET, here are some hints as to how to compile the sample program. The procedure is basically the same for any other C++ application that uses ANN. (Please note that I am not an experienced Windows programmer, so please consult your system documentation if this does not work for you.)

If you have downloaded the entire ANN distribution, you can certainly compile the sample program by opening the project file MS_Win32\sample\sample.vcproj. This assumes that that you have the full ANN distribution and have already compiled the ANN library.

It is possible to compile an application that uses ANN with just the following three elements, which are available from the "Precompiled files for for users of Microsoft Windows 2000" link on the ANN web page.

**Include files:** The directory ANN containing the three ANN include files, ANN.h, ANNx.h, and ANNperf.h,

**Library file:** ANN.lib,

**dll file:** ANN.dll.

Let us now illustrate how to compile the sample program from scratch. First, you will need to download the ANN full distribution, and make a copy of the file sample\ann_sample.cpp.

**Copy Files:** First, copy all the above files to a location that suits your preferences. For the sake of illustration, we will make the following assumptions in the subsequent presentation, but you may use whatever directories you like (in fact, they can all just be the same directory).

- **Source file:** Copy the file ann_sample.cpp to a directory containing your project source files, say, C:\My Sources.
- **Include files:** Copy the contents of the directory ANN, which contains the three ANN include files, to a directory where your include files are stored, say, C:\My Includes. (Alternatively, you can store this in the default directory where the linker looks for standard libraries, something like C:\Program Files\Microsoft Visual Studio .NET 2003\Vc7\include.)
- **Lib file:** Copy the file ANN.lib to a directory where your library files are stored, say, C:\My Libs. (Alternatively, you can store this in the default directory where the linker looks for standard libraries, something like, C:\Program Files\Microsoft Visual Studio .NET 2003\Vc7\lib.)
- **DLL file:** Copy the file ANN.dll to a directory where your DLL files are stored, say, C:\My DLLS. (Alternatively, you can store this in the system directory on your path environment variable, say, C:\WINDOWS\system32.)

**Create a New Project:** Open Visual Studio .NET and select the command "New Project." Select the appropriate project type. For ann_sample, this will be "WIN32 Console Project." Enter the desired project name (say, "ann_sample") and enter the path name of the directory where you would like the project files to be stored. (This may just be the same directory that contains ann_sample.cpp sources.)

**Add the Source:** Select the menu option "Project" → "Add Existing Item" and use the browser window to select your copy of the file ann_sample.cpp.

**Location of the include files:** In the "Solution Explorer" window, find the entry for your project name (say, "ann_sample") and right click and select "Properties." From the resulting pop-up

window select “C/C++” → “General.” Locate the field named “Additional Include Directories” and enter the full path name of the directory into which you copied the directory ANN, say, “C:\My Includes”. (If you chose to copy this directory to the default include directory, this is not necessary.)

**Location of the Library:** In the “Solution Explorer” window, window, find the entry for your project name (say, “ann_sample”) and right click and select “Properties.” From the resulting pop-up window select “Linker” → “General.” Locate the field named “Additional Library Directories” and enter the full path name of the directory where you stored ANN.lib, say, “C:\My Libs”. (If you chose to copy this file to the default library directory, this is not necessary.)

**Location of the DLL:** The system searches the directories whose names appear in the *Path* environment variable for the locations of DLL files. If you have chosen to store ANN.dll in your WINDOWS\system32 directory, then you not need to do anything more, since this will be searched. If you stored the file in a different directory, then you need to add this name to your Path variable. To do this, first open the Control Panel and select “System” (under “Performance and Maintenance”). Click on the “Advanced” tab and select “Environment Variables”. Find the variable “PATH” or “Path” (either under “System variables” or “User variables”). If it does not exist, then add it and enter in it the full path name of the directory where you stored the file ANN.dll, for example “C:\My Libs”. If it already exists, click the variable name to highlight it, and then click the “Edit” button. At the end of this string append a semicolon (“;”) followed by the above path name.

**Compile your program:** To compile the program return to Visual Studio .NET and select the menu command “Build” → “Build Solution.” (It should not produce any error messages, but if it complains about not being able to find ANN.h then recheck the above step about include files. If it complains about not being able to find ANN.lib then recheck the above step about the library file.)

At this point you should be able to execute the program. To do this, open a Command Prompt window, and go to the directory containing the executable file ann_sample.exe. Then enter ann_sample. It should print the program’s usage message. (If it complains that ANN.dll could not be found, recheck the above step about DLL files.) This is not very interesting. If you copy the files sample\data.pts and sample\query.pts from the full ANN distribution into this directory, then the sample program can be run using the command:

```
ann_sample -df data.pts -qf query.pts
```

## 2.2 Configuring ANN

In this section we describe a number of methods for configuring ANN. These include dealing with different distance measures, altering the types of coordinates, and dealing with the issue of whether a point can be its own nearest neighbor. It was felt that these features are typically set once for any given application, and that it would significantly complicate the library to allow support all possible combinations of settings. For this reason, these changes are made by altering ANN’s main header file include/ANN/ANN.h. Once set, they cannot be changed without recompiling the entire library.

### 2.2.1 Norms and Distances

ANN computes the distance between two points $p$ and $q$ as the length of the vector difference

$$p - q = (p_0 - q_0, p_1 - q_1, \ldots, p_{d-1} - q_{d-1}).$$

ANN's internal structure is oriented towards this type of distance, and it does not support, nor can it be easily modified, to handle other types of similarity measures, e.g., the cosine similarity measure, which based on the inner products of two vectors.

ANN employs a simple (but not elegant) method for computing vector lengths, which allows all the Minkowski norms. Given a vector $v$, and positive integer $k$, define the *Minkowski $L_k$ norm* to be

$$\|v\|_k = \left( \sum_{0 \le i < d} |v_i|^k \right)^{1/k}.$$

The familiar Euclidean norm is just the $L_2$ norm, and is ANN's default. The Manhattan norm is the $L_1$ distance. The max norm is the limiting case as $k$ tends to infinity

$$\|v\|_\infty = \max_{0 \le i < d} |v_i|.$$

As mentioned earlier, for the purposes of comparing the relative sizes of distances, it is not necessary to compute the final power of $1/k$, and so ANN does not do so. With some abuse of notation, we refer to the resulting quantity as the *squared norm* (even though squaring is only applicable to the Euclidean norm).

In general, ANN assumes that the distance between points $p$ and $q$ is computed as the length of the difference vector $v = p - q$, by the following formula:

$$\|v\| = root(pow(v_0) \oplus pow(v_1) \oplus \cdots \oplus pow(v_{d-1})),$$

where $root()$, $pow()$ are unary functions and $\oplus$ is any associative and commutative binary operator. For example, in the default case of the Euclidean norm, $pow(x) = x^2$, $x \oplus y = x + y$ and $root(x) = \sqrt{x}$. It is assumed that $pow()$ takes an argument of type ANNcoord and returns an ANNdist. The operator $\oplus$ works on objects of type ANNdist, and $root()$ takes an argument of type ANNdist and returns an object of type double. ANN does not compute the $root()$ function.

There is one more wrinkle in modifying distance computations. To speed-up the internal distance computations in nearest neighbor searching in high dimensional space, ANN uses a technique called *incremental distance calculation* [AM93a]. In incremental distance calculation, we are given two vectors $u$ and $v$, which differ only in one known coordinate, $i$, such that $|u_i| < |v_i|$. Further, suppose we already know $\|u\|$. We can compute $\|v\|$ in any Minkowski norm with only an additional constant number of computations (independent of the dimension). For this, we need a binary operator $\ominus$, which behaves like an inverse for the $\oplus$ operator. Then

$$\|v\| = \|u\| \oplus (pow(u_i) \ominus pow(v_i)).$$

When $\oplus$ is addition, then we define $\ominus$ to be subtraction. In the case of the $L_\infty$ norm, where $\oplus$ is max, there is no inverse for $\oplus$. But we can exploit the facts that for this metric, $pow$ is absolute value, and $|u_i| \le |v_i|$ and define $x \ominus y = y$, to achieve the desired result.

The main header file, include/ANN/ANN.h, contains a set of macros which control the computation of norms. They are ANN_POW, ANN_SUM (for $\oplus$), ANN_ROOT, and ANN_DIFF (for $\ominus$). The following table shows the values of these functions and corresponding macros for the Minkowski norms.

| Function | Macro | $L_1$ | $L_2$ | $L_p$ | $L_\infty$ |
|---|---|---|---|---|---|
| $pow(x)$ | ANN_POW(x) | $\|x\|$ | $x^2$ | $\|x\|^p$ | $\|x\|$ |
| $x \oplus y$ | ANN_SUM(x,y) | $x + y$ | $x + y$ | $x + y$ | $\max(x, y)$ |
| $root(x)$ | ANN_ROOT(x) | $x$ | $\sqrt{x}$ | $x^{1/p}$ | $x$ |
| $x \ominus y$ | ANN_DIFF(x,y) | $y - x$ | $y - x$ | $y - x$ | $y$ |

To change the norm, make the appropriate changes in the header file, and recompile the library.

### 2.2.2 More on Points, Cordinates, and Distances

One of the basic issues in providing a general purpose library for nearest neighbor searching is that of providing a method for defining the types used for point coordinates and distances. One way to do this would be through the use of templates in C++ (as is done in CGAL [Ove96]) or by replicating all of the basic procedures (as is done in OpenGL [Boa93]). In ANN we took the simpler, but less flexible approach of making this a configuration parameter. As a consequence, ANN can support any combination of coordinates and distances, but only one for any given compilation of the library.

There are two data types defined in the file include/ANN/ANN.h which are used to define these types. These are the data type for point coordinates, ANNcoord, and the type for squared distances, ANNdist. Their default values are:

```
typedef double  ANNcoord;              // coordinate data type
typedef double  ANNdist;               // distance data type
```

In general, ANNcoord may be set to any signed numeric type: char, short, int, long, float, double. (The reason for requiring that coordinates be signed is that the program computes differences of numbers of unknown relative magnitudes.)

The type ANNdist may be set to the desired type to hold squared distances. Observe that ANNdist should generally be of an equal or stronger type than ANNcoord. That is, if ANNcoord is set to int, then ANNdist should be either int, long, float, or double. ANN does not check for overflows in squared distance computations.

ANN assumes that there are two constant values defined in include/ANN/ANN.h. These are ANN_DBL_MAX, which stores the maximum possible double value and ANN_DIST_INF, which stores the maximum possible (squared) distance value. On most systems, ANN_DBL_MAX is assigned to DBL_MAX, which is defined in one of the C++ standard include files ⟨limits.h⟩ or ⟨float.h⟩.[2] The value of ANN_DIST_INF depends on the definition of ANNdist. For example, if ANNdist is double, then ANN_DIST_INF can be set to ANN_DBL_MAX. If it is long, then the maximum long value (e.g., LONG_MAX) can be used.

[2]Some older systems do not have these include files. If so, the library should be compiled with the option -DANN_NO_LIMITS_H, and an appropriate substitute should be provided in include/ANN/ANN.h.

The routine that dumps a tree needs to know roughly how many significant digits there are in a ANNcoord, so it can output points to full precision. This is defined as ANNcoordPrec. Its value needs only be set of ANNcoord is a floating-point type. All of these entities are defined in include/ANN/ANN.h.

### 2.2.3 Self Matches

In some applications of nearest neighbor searching, the nearest neighbor of a point is not allowed to be the point itself. That is, the nearest neighbor is defined to be the point of strictly positive distance that is closest to the query point. This occurs, for example, when computing the nearest neighbor from each point in a set to a different point in the same set. By setting the parameter ANN_ALLOW_SELF_MATCH in the file include/ANN/ANN.h to ANNfalse and then recompiling the package, the nearest neighbor must be of strictly positive distance from the query point. By default this parameter is set to ANNtrue, allowing self matches.

## 2.3 Modifying the Search Structure

One of the goals of ANN is to provide a testbed for various data structures for approximate and exact nearest neighbor searching. ANN provides a number of search structures, all within a common framework. For optimum performance and speed, it is sometimes advantageous to modify the search structure, depending on the nature of the data point or query point distributions. Before describing these changes, it is important to understand a bit of how the basic data structures operate.

### 2.3.1 ANN Data Structures

The two main data structures that ANN supports are *kd-trees* [Ben90, FBF77] and *box-decomposition trees* (or bd-trees for short) [AMN+98]. Let us discuss each in greater detail.

The kd-tree data structure is based on a recursive subdivision of space into disjoint hyperrectangular regions called *cells*. (See Fig. 2.) Each node of the tree is associated with such region $B$, called a *box*, and is associated with a set of data points that lie within this box. The root node of the tree is associated with a bounding box that contains all the data points. Consider an arbitrary node in the tree. As long as the number of data points associated with this node is greater than a small quantity, called the *bucket size*, the box is split into two boxes by an axis-orthogonal hyperplane that intersects this box. There are a number of different *splitting rules*, which determine how this hyperplane is selected. We will discuss these in detail later.

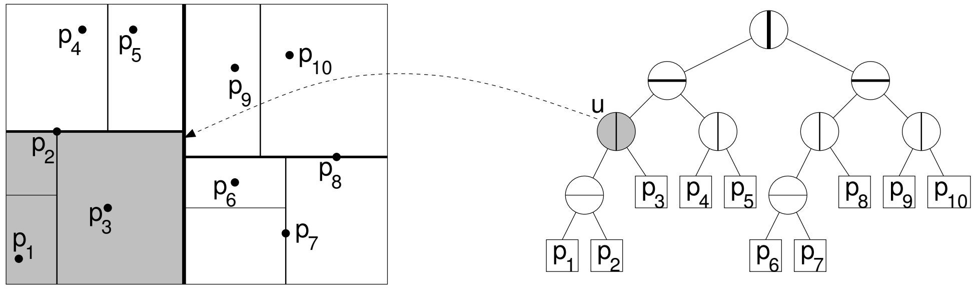


Figure 2: A kd-tree of bucket-size one and the corresponding spatial decomposition.

These two boxes are the cells associated with the two children of this node. The data points lying in the original box are split between these two children, depending on which side of the splitting hyperplane they lie. Points lying on the hyperplane itself may be associated with either child (according to the dictates of the splitting rule). When the number of points that are associated with the current box falls below the bucket size, then the resulting node is declared a *leaf node*, and these points are stored with the node.

The problem with the splitting operation used in kd-trees is that, when points are highly clustered, it may either take many splits to partition these points or the splits may result is highly elongated boxes (which can be problematic when searching is performed). ANN supports the bd-tree data structure to provide greater robustness for highly clustered data sets. The bd-tree is a variant of the data structure described in [AMN+98]. It differs from the kd-tree mainly in that, in addition to the splitting operation, there is a more general decomposition operation called *shrinking*. As above, consider a node whose associate box $B$ contains more points than the bucket size. A *shrinking rule* is then invoked. This rule may opt to simply defer and perform a split according to the prevailing splitting rule. Otherwise, it selects an axis-aligned box $B'$ lying within $B$. The points lying inside $B'$ are associated with one child and the points lying outside $B'$ are associated with the other child. As before, points lying on the boundary of $B'$ may be associated with either child.

Thus, in addition to the data points themselves, a kd-tree is specified by two additional parameters, the bucket size and a splitting rule. A box-decomposition tree is defined by these two parameters as well, and an additional parameter, the shrinking rule. ANN provides a number of different splitting rules and shrinking rules. It also provides reasonable default values for these parameters.

The (almost) complete prototypes for the constructors for kd-trees and bd-trees are given below. Refer back to Section 2.1.2 for definitions of the data point array $pa$, number of points $n$, and dimension $d$. The types ANNsplitRule and ANNshrinkRule are splitting rules and shrinking rules. They are described in later sections.

```
enum ANNsplitRule {                      // splitting rules for kd-trees
        ANN_KD_STD,                      // standard kd-splitting rule
        ANN_KD_MIDPT,                    // midpoint split
        ANN_KD_FAIR,                     // fair-split
        ANN_KD_SL_MIDPT,                 // sliding midpoint split
        ANN_KD_SL_FAIR,                  // sliding fair-split
        ANN_KD_SUGGEST};                 // the authors' suggestion for best

enum ANNshrinkRule {                     // shrinking rules for bd-trees
        ANN_BD_NONE,                     // no shrinking at all (just kd-tree)
        ANN_BD_SIMPLE,                   // simple splitting
        ANN_BD_CENTROID,                 // centroid splitting
        ANN_BD_SUGGEST};                 // the authors' suggested choice

ANNkd_tree(                              // build a kd-tree from a point array
        ANNpointArray   pa,              // point array
        int             n,               // number of points
        int             d,               // dimension
        int             bs = 1,          // bucket size
        ANNsplitRule    split = ANN_KD_SUGGEST);        // splitting rule
```

```
ANNbd_tree(                                     // build a bd-tree from a point array
        ANNpointArray   pa,             // point array
        int             n,              // number of points
        int             d,              // dimension
        int             bs = 1,         // bucket size
        ANNsplitRule    split  = ANN_KD_SUGGEST,        // splitting rule
        ANNshrinkRule   shrink = ANN_BD_SUGGEST);       // shrinking rule
```

### 2.3.2 Internal Tree Structure

Before discussing the various splitting and shrinking rules, we digress to discuss the internal structure of these two trees. This material is not needed for using the data structures, but it provides some background for understanding how the search algorithms work.

The root structure for both kd- and bd-trees contains the same information. It contains global information such as the number of points $n$, the dimension of the space $d$, and the bucket size, $b$. It does not store the points, but rather maintains a pointer to the array $pa$ of data points given in the constructor. It allocates an $n$-element array $pidx$ of point indices. It also stores a bounding hyperrectangle that contains all the data points, and a pointer to the root of the kd- or bd-tree.

A leaf node in either type of tree stores the number of points that are associated with this node (which ranges from 0 up to the bucket size) and an array of point indices. This point index array is actually a subarray of $pidx$.

A splitting node in a kd-tree contains the following information. First, it stores the integer *cutting dimension* (from 0 to $d-1$) indicating the coordinate axis that is orthogonal to the cutting hyperplane. Second it stores the *cutting value* where this plane intersects this axis. It also contains the two pointers to the left and right children (containing points lying to the low and high side of the cutting plane, respectively). The search algorithm uses a technique called *incremental distance computation* [AM93a] for speeding up the computation of distances between the query point and the associated cells of nodes of the tree. In order to implement incremental distance computation, it also stores two associated pieces of information with the cell. Consider the two hyperplanes bounding the cell that are parallel to the cutting plane. It stores the values at which these two hyperplanes intersect the cutting dimension's axis.

Leaf and splitting nodes in a bd-tree contain exactly the same information as in the kd-tree. The only difference is that the tree may also contain shrinking nodes. A shrinking node consists of a set of at most $2d$ orthogonal halfspaces. Each orthogonal halfspace is represented by storing a cutting dimension, a cutting value (which together define an orthogonal hyperplane), and an integer $-1$ or $+1$, which indicates to which side (negative or positive, respectively) of this plane the halfspace lies. The node contains the number of orthogonal halfspaces bounding the inner box, and an array of these halfspaces. (The reason for not storing all $2d$ bounding halfspaces is that in high dimensional applications, the shrinking node may only involve a relatively small number of halfspaces.)

### 2.3.3 Splitting Rules for kd-trees

This section describes the various splitting rules for kd-trees that are supported by ANN. Let $S$ denote the current subset of data points to be stored in the tree, let $n$ denote the number of points in $S$, let $C$ denote the current cell. Let $R(C)$ denote the bounding rectangle for the current cell.

The points of $S$ are all contained in $R(C)$. Let $R(S)$ denote the bounding rectangle for $S$. This rectangle is contained within (and may be equal to) $R(C)$. Initially, $S$ is the set of all data points, and $C$ is the root of the kd-tree, and $R(C)$ is the enclosing bounding rectangle for $S$. Define the *aspect ratio* of a rectangle to be the ratio between its longest and shortest side lengths. Given a dimension, define the *point spread* of $S$ along this dimension to be the difference between the largest and smallest coordinate for this dimension. This is equivalent to the longest side of $R(S)$.

Given a set of numbers $A$, a *median partition* of the $n$ numbers is a partition of $A$ into two subsets, one with $\lfloor n/2 \rfloor$ elements whose values are no greater than the median of $A$, and the other with $\lceil n/2 \rceil$ elements whose values are no less than the median of $A$.

ANN_KD_STD: This is called the *standard kd-tree splitting rule.* The splitting dimension is the dimension of the maximum spread of $S$. The splitting point is the median of the coordinates of $S$ along this dimension. A median partition of the points $S$ is then performed. This rule guarantees that the final tree has height $\lceil \log_2 n \rceil$, and size $O(n)$, but the resulting cells may have arbitrarily high aspect ratio.

ANN_KD_MIDPT: This is a simple splitting rule, which guarantees that cells have bounded aspect ratio, and is called the *midpoint splitting rule.* It simply cuts the current cell through its midpoint orthogonal to its longest side. It can be seen as a binary variant of a quad tree, since with every $d$ levels of the tree, a hypercube of side length $x$ is partitioned into equal hypercubes of side length $x/2$ each. If there are ties, it selects the dimension with the maximum point spread.

This rule can produce *trivial splits*, meaning that all of the points of $S$ lie to one side of the splitting plane. As a result, the depth of and size of the resulting tree can be arbitrarily large, and both may even exceed $n$ if the points are highly clustered.

ANN_KD_SL_MIDPT: This is a simple modification of the midpoint splitting rule, called the *sliding-midpoint rule.* It first attempts to perform a midpoint split, by the same method described above. If points of $S$ lie on both sides of the splitting plane then the algorithm acts exactly as it would for the midpoint split.

However, if a trivial split were to result, then it attempts to avoid this by "sliding" the splitting plane toward the points until it encounters the first data point. More formally, if the split is performed orthogonal to the $i$th coordinate, and all the points of $S$ have $i$-coordinates that are larger than that of the splitting plane, then the splitting plane is translated so that its $i$th coordinate equals the minimum $i$th coordinate among all the points of $S$. Let this point be $p_1$. Then the points are partitioned with $p_1$ in one part of the partition, and all the other points of $S$ in the other part. A symmetrical rule is applied if the points all have $i$th coordinates smaller than the splitting plane.

This rule cannot result in any trivial splits, implying that the tree has maximum depth $n$ and size $O(n)$. It is possible to generate a cell $C$ of very high aspect ratio, but it can be shown that if it does, then $C$ is necessarily adjacent to a sibling cell $C'$ that is fat along the same dimension that $C$ is skinny. It turns out that cells of high aspect ratio are not problematic for nearest neighbor searching if this occurs.

ANN_KD_FAIR: This is a compromise between the standard kd-tree splitting rule (which always splits data points at their median) and the midpoint splitting rule (which always splits a box

through its center. It is called the *fair-split rule.* The goal of this rule is to achieve as nicely balanced a partition as possible, provided that the aspect ratio bound is never violated.

A constant FS_ASPECT_RATIO = 3 is defined in the source file src/kd_split.cpp. Given a cell, it first determines the sides of this cell that can be split in some way so that the ratio of the longest to shortest side does not exceed FS_ASPECT_RATIO are identified. Among these sides, it selects the one in which the points have the largest spread. It then splits the points in the most even manner possible, subject to maintaining the bound on the ratio of the resulting cells. To determine that the aspect ratio will be preserved, we determine the longest side (other than this side), and determine how narrowly we can cut this side, without causing the aspect ratio bound to be exceeded.

This procedure is more robust than either the kd-tree splitting rule or the pure midpoint splitting rule when data points are highly clustered. However, like the midpoint splitting rule, if points are highly clustered, it may generate a large number of trivial splits, and may generate trees whose size exceeds $O(n)$.

ANN_KD_SL_FAIR: This rule is called *sliding fair-split.* It is a splitting rule that is designed to combine the strengths of both fair-split with sliding midpoint split. Sliding fair-split is based on the theory that there are two types of splits that are good: balanced splits that produce fat boxes, and unbalanced splits provided the cell with fewer points is fat.

This splitting rule operates by first computing the longest side of the current bounding box. Then it asks which sides could be split and still satisfy the aspect ratio bound with respect to this side. Among these, it selects the side with the largest spread (just as fair-split would). It then considers the most extreme cuts that would be allowed by the aspect ratio bound. This is done by dividing the longest side of the box by the aspect ratio bound. If the median cut lies between these extreme cuts, then we use the median cut. If not, then consider the extreme cut that is closer to the median. If all the points lie to one side of this cut, then we slide the cut until it hits the first point. This may violate the aspect ratio bound, but will never generate empty cells. However the sibling of every such skinny cell is fat, as with sliding midpoint split.

ANN_KD_SUGGEST: This is the default splitting rule. It is set to the splitting rule which, in the authors' opinion, performs best with typical data sets. It is currently set to ANN_KD_SL_MIDPT, the sliding midpoint rule.

### 2.3.4 Shrinking Rules for bd-trees

This section describes the various shrinking rules for bd-trees that are supported by ANN. Let $S$, $C$, and $n$, be the same as defined in the previous section and let $d$ denote the dimension

ANN first attempts to perform shrinking. Each of shrinking rules have the option of electing to not shrink. If this is the case, then processing is passed to the current splitting rule instead.

ANN_BD_NONE: When this rule is selected, no shrinking is performed at all, only splitting. A bd-tree generated with this shrinking rule is identical to a kd-tree.

ANN_BD_SIMPLE: This is called a *simple shrink.* It depends on two constants: BD_GAP_THRESH whose value is 0.5, and BD_CT_THRESH whose value is 2. It first computes the tight bounding

box of the points, and computes the $2d$ *gaps*, that is, the distances between each side of the tight enclosing rectangle for the data points $R(S)$ and the corresponding side of the cell's bounding rectangle $R(C)$. If at least BD_CT_THRESH of the gaps are larger than the length the longest side of $R(S)$ times BD_GAP_THRESH, then it shrinks all sides whose gaps are at least this large. After the shrink has been performed, all of the points of $S$ lie in the inner box of the shrink, and the outer box contains no points. If none of the gaps is large enough, then no shrinking is performed, and splitting is performed instead.

ANN_BD_CENTROID: This rule is called a *centroid shrink*. Its operation depends on two constants: BD_MAX_SPLIT_FAC and BD_FRACTION, both of whose value are 0.5. It repeatedly applies the current splitting rule (without actually generating new cells in the tree). In each case we see which half of the partition contains the larger number of points, and we repeat the splitting on this part. This is repeated until the number of points falls below a fraction of BD_FRACTION of the original size of $S$. If it takes more than dim*BD_MAX_SPLIT_FAC splits for this to happen, then we shrink to the final inner box. All the other points of $S$ are placed in the outer box. Otherwise, shrinking is not performed, and splitting is performed instead.

ANN_BD_SUGGEST: This is the default shrinking rule. It is set to the shrinking rule which, in the authors' opinion, performs best in most circumstances. It is currently set to ANN_BD_SIMPLE.

## 2.4 Search Algorithms

ANN supports two different methods for searching both kd- and bd-trees. The first is called *standard search*. It is the simpler of the two methods, and visits cells in order based on the hierarchical structure of the search tree. The second method, called *priority search*, visits cells in increasing order of distance from the query point, and hence, should converge more rapidly on the true nearest neighbor. However, priority search requires the use of a heap, and so has a somewhat higher overhead. When the error bound is small, standard search seems to be slightly faster. When larger error bounds are used, or when early termination is used (described in Section 2.4.3 below) then priority search seems to be superior.

### 2.4.1 Standard Search

Standard search is an adaptation of the search algorithm described by Friedman, Bentley, and Finkel [FBF77], but for approximate nearest neighbor searching. The algorithm operates recursively. When first encountering a node of the kd-tree the algorithm first visits the child that is closest to the query point. On return, if the box containing the other child lies within $1/(1+\epsilon)$ times the distance to the closest point seen so far, then the other child is visited recursively. The distance between a box and the query point is computed exactly (not approximated), using incremental distance updates, as described by Arya and Mount [AM93a].

The search is similar for bd-trees. For shrinking nodes we compute the distances to the inner box and the outer box and recurse on the closer. In case of a tie, we favor the inner box.

The prototype is given below. The argument $q$ is the query point, $k$ is the number of nearest neighbors, to return *nn_idx* is an array of at least $k$ point indices, and *dists* is an array of at least $k$ elements of type ANNdist, and *eps* is the (optional) error bound in nearest neighbor searching. If *eps* is omitted or zero, then the $k$ nearest neighbors are computed exactly. The $k$ (approximate) nearest neighbor squared distances are stored in the elements of *dists*, and the indices of the nearest

neighbors are returned in the elements of *nn_idx*. An error results if $k$ exceeds the number of data points. The next two sections describe the search algorithms in greater detail.

```
virtual void annkSearch(           // standard search
    ANNpoint        q,             // query point
    int             k,             // number of near neighbors to return
    ANNidxArray     nn_idx,        // nearest neighbor array (returned)
    ANNdistArray    dists,         // dists to near neighbors (returned)
    double          eps=0.0);      // error bound
```

### 2.4.2 Priority Search

Priority search is described by Arya and Mount [AM93a]. The cell of the kd-tree containing the query point is located, and cells are visited in increasing order of distance from the query point. This is done as follows. Whenever we arrive at a nonleaf node, we compute the distances from the query point to the cells of the two children. We enqueue the further child on a priority queue, sorted by distance, and then visit the closer child recursively. On arriving at a leaf node, we compute the distances to the points stored in this node, and continue by dequeing the next item from the priority queue. The search stops either when the priority queue is empty (meaning that the entire tree has been searched) or when the distance to the nearest cell on the priority queue exceeds the distance to the nearest point seen by a factor of more than $1/(1+\epsilon)$.

The search is similar for bd-trees. For shrinking nodes we compute the distances to the inner box and to the outer box. We visit the closer one and enqueue the further, breaking ties in favor of the inner box. (Note that this differs slightly from the description in [AMN$^+$98]. There the cell of the outer child is the set theoretic difference between the inner box and the outer box, implying a more complex distance computation.) The prototype for both trees is essentially the same as that of the standard search, described above.

A priority-search variant of annkSearch() is provided, and the prototype is given below. There is no priority-search variant of the fixed-radius search, annkFRSearch().

```
virtual void annkPriSearch(        // priority search
    ANNpoint        q,             // query point
    int             k,             // number of near neighbors to return
    ANNidxArray     nn_idx,        // nearest neighbor array (returned)
    ANNdistArray    dists,         // dists to near neighbors (returned)
    double          eps=0.0);      // error bound
```

### 2.4.3 Early Termination Option

In situations where response time is critical, the search algorithm (either standard or priority search) can be forced to terminate prior to the normal termination conditions described earlier. This is done by invoking the function annMaxPtsVisit(). The single integer argument is the maximum number of points that the search algorithm will visit before terminating. The algorithm maintains a count of the number of points visited, and tests this quantity before visiting each leaf node. Since the test is only made prior to visiting each leaf, it may visit some additional points depending on the bucket size.

The bound remains in effect until it is changed by a subsequent call to this function. To disable early termination, call the function with the argument 0. This is not a member function, but a

free-standing procedure, which sets a global variable. Thus, if there are two search structures to which queries are being performed, and different maximum point values are desired for each, then it would be necessary to call this function prior to switching between the structures.

```
void annMaxPtsVisit(
    int                 maxPts);
```

If this bound is set, the neraest neighbor search procedures may terminate before all of the $k$ nearest neighbors have been encountered. Therefore, this function should be invoked with care.

## 2.5 Search Tree Utility Functions

ANN provides a number of utility member functions for gathering statistics on kd-trees and bd-trees, printing the tree in a format suitable for reading, and dumping the tree for the purpose of reading it by another program. Since they are member functions, they are invoked in essentially the same way as is the annkSearch procedure.

### 2.5.1 Printing the Search Structure

The procedure Print is used to print the contents of a kd- or bd-tree in a format that is suitable for reading. This is provided primarily for debugging purposes. This procedure prints the nodes of the tree according to a right-to-left inorder traversal. That is, for an internal node it first prints the high (or outer) child, then the node itself, then the low (or inner) child. (This is done this way because by tilting the output clockwise, the traversal appears to be left to right.)

For each leaf, it prints the number of points associated with the leaf, and for each point it outputs the index of the point. For each splitting node, it outputs the cutting dimension (*cd*), the cutting value (*cv*), the low bound (*lbnd*), and the high bound (*hbnd*) for the node. For shrinking nodes it prints the set of bounding halfspaces, two per line. For each bounding halfspace it prints the cutting dimension, the cutting value, and indicates whether the inner box lies on the greater or lesser side of the cutting value.

The level of the node in the tree is indicated by its indentation in the output. If the boolean *with_pts* argument is true, then the data coordinates points are printed before the tree, otherwise they are not printed. The other argument is the output stream to which the output is sent.

```
void Print(                         // print the tree (for debugging)
    ANNbool         with_pts,       // print points as well?
    ostream&         out);          // output stream
```

### 2.5.2 Saving and Restoring the Search Structure on a File

The member function Dump() copies a kd- or bd-tree and the associated points to a stream. (It is currently not provided from the brute-force data structure.) The tree is printed in preorder, first printing each node, then the low (or inner) child, followed by the high (or outer child). This output format is not intended for human reading, but instead for saving the data structure for the purposes of loading it later, or for consumption by another program (such as ann2fig), for analysis of the tree.

As with Print, the first argument indicates whether the points are to be printed, and the second argument is the output stream to which the output is sent.

```
void Dump(                          // dump entire tree
    ANNbool         with_pts,       // print points as well?
    ostream&         out);          // output stream
```

To restore a saved structure, there is a constructor, which is given an input stream, which is assumed to have been created through the Dump() function, and creates a search structure from the contents of this file. It is assumed that the file was saved with the points printed.

```
ANNkd_tree(                         // build structure from a dump file
    istream&         in);           // input stream for dump file
```

For example, to save the current structure the_tree to ostream foo and then load a new structure from istream bar the following could be used.

```
ANNkd_tree* the_tree = new ANNkd_tree(...);
the_tree->Dump(ANNtrue, foo);       // save the current tree contents
delete the_tree;                    // deallocate (to prevent memory leaks)
// ... (sometime later)
the_tree = new ANNkd_tree(bar);     // restore a copy of the old tree
```

The dump output format consists of the following elements. All entries on a line are separated by white space.

**Header:** A line containing #ANN followed the version number for ANN.

**Points:** If the parameter *with_pts* is true, then this section is printed. It starts with a line beginning with the word points, followed by the dimension of the space $d$, and the number of points $n$. This is then followed by $n$ lines, each containing the integer index of the point, followed by the $d$ coordinates of the point. One issue for floating point data is the desired output precision. This is stored in the variable ANNcoordPrec in the file include/ANN/ANN.h.

**Tree Structure:** The tree structure describes the tree contents. It begins with a single header entry followed by a series of entries, one for each node of the tree, given according to a preorder traversal of the tree, as described above. There is no distinction made between kd-trees and bd-trees, except that kd-trees have no shrinking nodes.

**Header:** This starts with a line containing the word tree, followed by the dimension $d$, the number of points $n$, and the bucket size $b$. This is followed by 2 lines, giving the lower endpoint and upper endpoint of the bounding box of the tree's root cell.

**Leaf node:** The starts with the word leaf followed by the number of points $m$ stored in this leaf (from 0 to $b$), followed by the $m$ integer indices of these points.

**Splitting node:** Starts with the word split followed by the cutting dimension, cutting value, lower bound and upper bound for the node.

**Shrinking node:** Starts with the word shrink followed by the number of bounding sides $m$, followed by $m$ lines, each containing a cutting dimension, cutting value, and the side indicator (1 or $-1$), for this bounding side.

### 2.5.3 Gathering Statistics on Tree Structure

The procedure getStats computes statistics on the shape and properties of the search tree. These are returned in the structure ANNkdStats. This is something of a misnomer, because the stats apply to both kd-trees and bd-trees. This structure is returned (as the argument) to the following procedure.

```
void getStats(                          // compute tree statistics
    ANNkdStats&      st);               // the statistics (returned)
```

The statistics structure contains the following public members, which are all set by the call to getStats. The total number of leaves is stored in n_lf. In order to save space, ANN distinguishes between leaves that do and do not contain points. Leaves that contain no points are called *trivial leaves*, and are counted in n_tl. (Because some splitting rules may generate a large number of trivial leaves, ANN saves space by storing just one *canonical* trivial leaf cell, and each trivial leaf generated by the algorithm is implemented by a single pointer to this canonical cell.)

Internal nodes are of two types, splitting nodes and shrinking nodes. The former is counted in n_spl and the latter in n_shr. Since kd-trees do not perform shrinking, the value of n_shr is nonzero only for bd-trees. The maximum depth of the tree is stored in depth. The *aspect ratio* of a leaf cell is the ratio between the lengths of its longest and shortest sides. There is theoretical evidence that nearest neighbor searching is most efficient when the aspect ratio is relatively small (say, less than 20). The average aspect ratio of all the leaf cells is stored in avg_ar.

```
class ANNkdStats {                      // stats on kd-tree
public:
    int dim;                            // dimension of space
    int n_pts;                          // number of points
    int bkt_size;                       // bucket size
    int n_lf;                           // number of leaves
    int n_tl;                           // number of trivial leaves
    int n_spl;                          // number of splitting nodes
    int n_shr;                          // number of shrinking nodes (bd-trees only)
    int depth;                          // depth of tree
    float avg_ar;                       // average leaf aspect ratio
}
```

## 2.6 Compile-Time Options

ANN contains a number of options that are activated when the program is compiled. These are activated or deactivated by adding these parameters to the "CFLAGS" variable in the file Make-config, before compiling the library. For example:

```
"CFLAGS = -O3 -Wall -DANN_PERF"
```

Some of these options are described below (see Make-config for others). By default, none of these are active, except the "-O" for optimization.

-O: (or "-O3") Produce code optimized for execution speed.

-DANN_PERF: This causes code to be compiled that performs additional performance measurements on the efficiency of the structure (e.g., the average CPU time per query). Setting this option can slow execution by around 10%.

-DANN_NO_LIMITS_H: Use this if your system does not have either of the include files ⟨limits.h⟩ or ⟨float.h⟩, which are needed for the definition of system-dependent quantities like DBL_MAX. (Also see include/ANN/ANN.h for other changes that may be needed.)

-DANN_NO_RANDOM: Use this if your system does not support the built-in pseudo-random number generators srandom() or random(). Pseudo-random number generation is used in the utility program test/ann_test. (It is not required if you just want to compile the ANN library.) Virtually all C++ systems provide a simple pseudo-random number in the form of two build-in functions srand() and rand(). However, these functions are inferior to the less widely available functions srandom() and random(). By setting this option, the program will use functions srand() and rand() instead. This is needed on most Microsoft Visual C++ systems.

# 3 Utility Programs

As mentioned earlier, ANN is not just a library for nearest neighbor searching, but it is also provides a testbed for nearest neighbor data structures and algorithms. There are two utility programs provided with ANN for the purpose of evaluating and visualizing search structures (under various splitting and shrinking rules) for a number of different data distributions. These are ann_test and ann2fig, described in the following sections. If you are just interested in the ANN library, you do not need to compile these two programs.

## 3.1 ann_test: A Test and Evaluation Program

The program ann_test provides a simple script language for setting up experiments involving ANN. It allows the user to generate data and query sets of various sizes, dimensions, and distributions, to build kd- and bd-trees of various types, and then run queries and outputting various performance statistics.

The program reads commands from the standard input and writes its results to the standard output. The input consists of a sequence of *directives*, each consisting of a directive name, followed by list of 0 or more arguments (depending on the directive). Arguments and directives are separated by white space (blank, tab, and newline). String arguments are not quoted, and consist of a sequence of nonwhite characters. The character "#" denotes a comment. The following characters up to the end of line are ignored. Comments may only be inserted between directives (not within the argument list of a directive).

Directives are of two general types: *operations*, which cause some action to be performed and *parameter settings*, which set the values of various parameters that affect later operations. As each line of the input file is read, the appropriate operation is performed or the appropriate parameter value is set. For example, to build a kd-tree with a certain splitting rule, first set the parameter that controls the splitting rule, and then invoke the operation for building the tree.

A number of the operations (particularly those involving data generation) involve generation of pseudo-random numbers. The pseudo-random number generator can be controlled by an integer *seed* parameter. Once the seed is set, the subsequent sequence of pseudo-random numbers is completely determined. For example, to generate identical but pseudo-random data point sets and query point sets, first set the seed to some desired value, issue the operation to generate the data points, then reset the seed to this same value, and then issue the operation to generate the query points.

The following section lists the basic operations (directives that perform actions), and the remaining sections describe various parameters that affect how these operations are performed. Arguments and their type are indicated with angled brackets. For example ⟨*string*⟩ means that the argument is a character string. String and filename arguments consist of a sequence of characters with no embedded whitespaces (blank, tab, or newline). No quoting is needed (nor is it allowed). Currently there is no facility for placing comments within the script file.

### 3.1.1 Operations

output_label ⟨*string*⟩: Prints the string to the output file.

gen_data_pts: Generates a set of pseudo-random data points. The number of points is determined by the data_size parameter, the number of coordinates is determined by the dim parameter,

and the point distribution is determined by the distribution parameter. Data points generated by an earlier call to this operation or the read_data_pts operation are discarded. Relevant parameters: data_size, dim, std_dev, corr_coef, colors, seed, and distribution.

gen_query_pts: Same as gen_data_pts but for the current query point set. Relevant parameters: data_size, dim, std_dev, corr_coef, colors, seed, and distribution.

read_data_pts ⟨*file*⟩: Reads a set of data points from the given file. Each point consists of a sequence of dim numbers (of type ANNcoord) separated by whitespace (blank, newline, or tab). It is assumed that the file contains no more points than given by the data_size parameter, otherwise only this many points of the data set are input. Relevant parameters: data_size, dim.

read_query_pts ⟨*file*⟩: Same as read_data_pts but for the current query point set. The parameter query_size indicates the maximum number of points. Relevant parameters: query_size, dim.

build_ann: Builds either a kd-tree or bd-tree for the current data point set according to current splitting rule (see the split_rule parameter) and shrinking rule (see shrink_rule). If the shrinking rule is "none" then a kd-tree results, and otherwise a bd-tree results. Relevant parameters: split_rule, shrink_rule.

run_queries ⟨*string*⟩: Apply nearest neighbor searching to the current query point set using the current approximate nearest neighbor structure (resulting from build_ann, for the current number of nearest neighbors (see near_neigh), and the current error bound (see epsilon), and with the search strategy specified in the argument. Possible strategies are:

standard: Standard search. (See Section 2.4.1.)

priority: Priority search. (See Section 2.4.2.)

Relevant parameters: epsilon, near_neigh, max_pts_visit.

dump ⟨*file*⟩: Dump the current search structure to the specified file. See Section 2.5.2. If the dump file is to be read by the ann2fig program, then the file name should end with the suffix ".dmp".

load ⟨*file*⟩: Loads a tree from a data file that was created by the dump operation. Any existing tree structure and data point storage will be deallocated.

### 3.1.2 Options Affecting Tree Structure:

split_rule ⟨*string*⟩: Type of splitting rule to use in building a kd-tree or bd-tree. Choices are as follows. See Section 2.3.3 for further information.

standard: Perform standard optimized kd-tree splitting.

midpt: Perform midpoint splitting.

fair: Perform fair-split splitting.

sl_midpt: Perform sliding midpoint splitting.

sl_fair: Perform sliding fair-split splitting.

suggest: Perform the authors' suggested choice for the best splitting rule. (Currently sl_midpt.)

Default: suggest.

shrink_rule ⟨*string*⟩: Specifies the type of shrinking rule to use in building a bd-tree data structure. If none is specified, then no shrinking is performed and the result is a kd-tree. Choices are as follows. See Section 2.3.4 for further information.

none: Perform no shrinking.

simple: Perform simple shrinking.

centroid: Perform centroid shrinking.

suggest: Perform the authors' suggested choice for best. (Currently none.)

Default: none.

bucket_size ⟨*int*⟩: Bucket size, that is, the maximum number of points stored in each leaf node of the search tree. Bucket sizes larger than 1 are recommended for the "midpt" splitting rule when the dimension is 3 or higher. It is also recommended when performance evaluation shows that there are a large number of trivial leaf cells. Default: 1.

### 3.1.3 Options Affecting Data and Query Point Generation

The test program generates points according to the following probability distributions.

uniform: Each coordinate is chosen uniformly from the interval $[-1, 1]$.

gauss: Each coordinate is chosen from the Gaussian distribution with the current standard deviation (see std_dev) and mean 0.

clus_gauss: Some number of cluster centers (set by the parameter colors below) are chosen from the above uniform distribution. Then each point is generated by selecting a random cluster center, and generating a point with a Gaussian distribution with the current standard deviation (see std_dev) centered about this cluster center.

Note that once the cluster centers have been set, they are not changed for the remainder of the execution of the program. This is done so that subsequent calls to generate points from a clustered distribution will be generated under the same distribution.

laplace: Each coordinate is chosen from the Laplacian distribution with zero mean and the standard deviation one.

**Correlated distributions:** The following two distributions have been chosen to model data from applications in speech processing. These two point distributions were formed by grouping the output of autoregressive sources into vectors of length $d$. It uses the following recurrence to generate successive outputs:

$$X_i = \rho X_{i-1} + W_i,$$

where $W_n$ is a sequence of zero-mean, independent, identically distributed random variables. The recurrence depends on three parameters, the choice of $X_1$, the choice of $W_i$, and the value of the correlation coefficient, $\rho$. For both of the distributions below, $\rho$, is set to the current value of the parameter corr_coef. The values of $X_1$ and $W_i$ are defined below. The first

component $X_1$ is selected from the corresponding uncorrelated distribution (either Gaussian or Laplacian) and the remaining components are generated by the above equation. $W_n$ is chosen by marginal density of $X_n$ is normal with the current standard deviation.

co_gauss: Both $X_1$ and $W_i$ are chosen from the Gaussian distribution with the current standard deviation (see std_dev) and mean 0.

co_laplace: Both $X_1$ and $W_i$ are chosen from the Laplacian distribution zero mean and the standard deviation one.

clus_orth_flats: This distribution consists of a set points clustered among a collection of lower dimensional flats (hyperplanes) within the hypercube $[-1, 1]^d$. A set of color orthogonal flats are generated each of whose dimension is a random integer between 1 and max_clus_dim. The points are evenly distributed among the clusters. For each cluster, we generate points uniformly distributed along the flat within the hypercube, and with a Gaussian error with standard deviation std_dev added. (See parameters color, std_dev, and max_clus_dim below.)

clus_ellipsoids: This distribution consists of a set points clustered among a collection of lower dimensional ellipsoids. A set of color clusters are generated each of which is centered about a point chosen uniformly from $[-1, 1]^d$. Each cluster has a dimension, which is selected randomly from 1 to max_clus_dim. For each of the unselected coordinates, the coordinate is centered around the cluster center with a Gaussian error with a standard deviation std_dev (which is presumably small). Otherwise, for the selected coordinates, a standard deviation for this coordinate and cluster is generated uniformly in the range [std_dev_lo, std_dev_hi]. The points are evenly distributed among the clusters. (See parameters color, std_dev, std_dev, std_dev, and max_clus_dim below.)

planted: In high dimensional spaces, interpoint distances tend to be highly clustered around the mean value. Approximate nearest neighbor searching makes little sense in this context, unless it is the case that each query point is significantly closer to its nearest neighbor than to other points. Thus, the query points should be planted close to the data points. Given a source data set, this procedure generates a set of query points having this property.

A source data array and a standard deviation are given. A random point is generated as follows. We select a random point from the source data set, and we generate a Gaussian point centered about this random point and perturbed by a normal distributed random variable with mean zero and the given standard deviation along each coordinate. (Note that this essentially the same a clustered Gaussian distribution, but where the cluster centers are given by the source data set.)

Here are the parameters that affect point generation and representation.

dim ⟨*int*⟩: The dimension of the space. (Warning: Once set, if this value is changed then new data points and query points should be generated.) Default: 2.

seed ⟨*int*⟩: Set the seed for future pseudo-random number generation. Default: 0.

data_size ⟨*int*⟩: The number of data points. When reading data points from a file, this indicates the maximum number of points for purposes of storage allocation. Default: 100.

query_size ⟨*int*⟩: The number of query points. When reading query data points from a file, this indicates the maximum number of points for purposes of storage allocation. Default: 100.

std_dev ⟨*float*⟩: The standard deviation (used in gauss, clus_gauss, and clus_orth_flats distributions). For the clus_ellipsoids distribution it is the small distribution for the unselected dimensions. Default: 1.

std_dev_lo ⟨*float*⟩: (and std_dev_hi) are the standard deviations for the selected dimensions in the clus_ellipsoids distribution. Default: 1.

corr_coef ⟨*float*⟩: The correlation coefficient (used in co_gauss and co_lapace distributions). Default: 0.05.

colors ⟨*int*⟩: The number of color classes (clusters) (used in the clus_gauss and clus_orth_flats distributions). Default: 5.

max_clus_dim ⟨*int*⟩: Maximum number of dimensions in each flat of the clus_orth_flats and clus_ellipsoids distributions. Default: 1.

distribution ⟨*string*⟩: The type of point distribution. These can be selected from the list given above. Default: uniform.

### 3.1.4 Options Affecting Nearest Neighbor Searching

epsilon ⟨*float*⟩: The relative error bound for approximate nearest neighbor searching. Default: 0.

near_neigh ⟨*int*⟩: The number of nearest neighbors to report. This must not exceed the number of data points, or an error will result. Default: 1.

max_pts_visit ⟨*int*⟩: The maximum number of points to visit before terminating. If set to zero, then the search runs until its natural termination condition is satisfied. Default: 0 (use the natural termination condition).

radius_bound ⟨*float*⟩: Sets an upper bound on the nearest neighbor search radius. If the bound is positive, then fixed-radius nearest neighbor searching is performed, and the count of the number of points in the range is returned. If the bound is zero, then standard search is used. This can only be used with standard, not priority, search. Default = 0 (uses standard search.)

### 3.1.5 Options Affecting the Amount of Output

The program has a various levels of output "verbosity." At the lowest level (silent) no output is generated, and at the highest level (show_struct) the contents of the tree, all results, and statistics are printed. The output levels grow monotonically, so that each subsequent level prints all the information from the previous levels.

stats ⟨*string*⟩: The parameter value may be any of the following.

silent: No output is generated.

exec_time: Prints the execution time in CPU seconds for queries.

prep_stats: Prints preprocessing statistics for the tree structure and time to construct are printed after the build_ann operation has been performed. This includes the CPU time to build the structure, the depth of the search tree and the number of various types of nodes in the tree.

query_stats: Prints statistics about the query processing after the run_queries operation has been performed. These are described in greater detail below.

query_res: Prints the results of the queries, that is, the indices of the $k$ nearest neighbors as reported by the algorithm after run_queries has been performed.

show_pts: This causes data points (or query points) to be printed after operations like gen_data_pts and read_data_pts have been performed.

show_struct: This causes the contents of the search tree to be printed after the operation build_ann has been performed.

#### 3.1.6 Options Affecting the Validation

One of the interesting aspects of approximate nearest neighbor searching is that the program typically produces average approximation errors that are much smaller (often by a factor of 10) than allowed by the error parameter eps.

This program can be used to measure the *average error* over a set of queries. This is done by first running the queries using the approximation algorithm and saving the results, and then running the queries using exact nearest neighbor searching (which is done by the brute-force data structure). The average error for a single reported nearest neighbor at distance $x$, is computed by first finding the distance $x^*$ to the corresponding true nearest neighbor. If $x = x^* = 0$ then the error is defined to be 0, and otherwise it is $(x - x^*)/x^*$. These errors are computed for all query points and over all $k$ nearest neighbors for each query point.

As part of the validation process, the program checks that no error exceeds eps, and issues an error message if this is violated.

The second type of error is called the *rank error*. Define the rank of the data point as the number of data points that are at least as close as this point is to the query point. Thus the nearest neighbor has rank 1 (assuming no other points are of equal distance). To compute the rank error of each result, we compute its rank $r$ among the true nearest neighbors that were computed. (If it is further from all the true nearest, then $r$ is true_nn $+ 1$.) If this point is reported as the $j$-th nearest neighbor (where the closest point is $j = 1$) then the rank error for this point is $\max(0, j - r)$. The rank error is computed for all query points and all the $k$ nearest neighbors for each query point.

validate ⟨*string*⟩: This enables validation. This is used for debugging and error analysis (described further below). When validation is enabled, some number of exact nearest neighbors (as indicated by the parameter true_near_neigh) are computed by a simple brute force algorithm, and average errors and rank errors are computed.

Because the brute-force algorithm is used, the validation process can take a very long time, especially for large data sets in high dimensional spaces. Valid arguments are: either on or off. Default: off.

true_near_neigh ⟨*int*⟩: This parameter indicates the number of true nearest neighbors to compute for the validation process. This information is used in computing rank errors and average

errors. Its value should be at least as high as the value of near_neighbor. The default value of this parameter is 10 more than the value of near_neigh, the number of nearest neighbors for each query.

Here is an annotated example of an input to the ann_test program. The comments to the right are not part of the file. The parameters have been indented to distinguish them from operations.

```
output_label test-run-0    # output a label for this run
  validate on              # enable validation
  dim 16                   # points in dimension 16
  stats query_stats        # output performance statistics for queries
  seed 121212              # pseudo-random number seed
  data_size 1000
  distribution uniform
gen_data_pts               # generate 1000 uniform data points in dim 16
  query_size 100
  colors 10
  std_dev 0.05
  distribution clus_gauss
gen_query_pts              # generate 100 query points in 10 clusters
  bucket_size 2
  split_rule fair
  shrink_rule none
build_ann                  # build a kd-tree with fair-split and bucket size 2
  epsilon 0.1
  near_neigh 5
  max_pts_visit 100        # stop search if more than 100 points seen
  true_near_neigh 20       # compute 20 nearest neighbors when validating
run_queries standard       # run queries; 5 near neighbors, allowing 10% error
  data_size 500
read_data_pts data.in      # read up to 500 points from file data.in
  split_rule sl_midpt
  shrink_rule simple
build_ann                  # build bd-tree; sliding midpoint and simple shrink
  epsilon 0
run_queries priority       # run the same queries; with no allowable error
```

### 3.2 ann2fig: Visualization Tool

The program ann2fig inputs an ANN dump file, as generated by the dump operation of the ann_test program, and (optionally) the coordinates of the data points, and generates a 2-dimensional display of the geometric structure of the search tree. The output form is called *fig format*, e.g., as used by the xfig program (Ver 3.1). The resulting fig file may then be displayed using xfig, or converted to any of a number of other formats using the Unix program fig2dev. An example is shown in Fig. 1 in Section 2.

If the points are in dimension 2, then the entire tree is displayed. If the dimension is larger than 2 then the user has the option of selecting any orthogonal 2-dimensional "slice" to be displayed. A slice is an axis-aligned 2-dimensional plane in $d$-dimensional space. We assume that dimensions are numbered from 0 to $d-1$. The slice is specified giving the two *plane dimensions* that correspond to the $x$- and $y$-dimensions of the final output, and then giving the values at which this plane cuts each

of the remaining coordinate axes, called *slicing values*. There are two ways to specify the slicing values. The first is by giving a real *default slicing value*, $z$, which means that the slicing plane cuts all the coordinate axes (other than the plane dimensions) at the value $z$. The other is by specifying a set of *slicing pairs* each consisting of a dimension $i$, $0 \le i < d$, and value $z$, which means that the plane cuts the $i$th coordinate axis at value $z$. For example, given a 4-dimensional space, with coordinates 0 through 3, the 2-dimensional plane $x_2 = 0.5$, $x_3 = -2.4$, could be specified by the slicing pairs $(2, 0.5)$ and $(3, -2.4)$.

Given the slicing plane, a leaf cell is either disjoint from the slicing plane or it intersects the slicing plane in a rectangle. In the former case, the leaf cell is not displayed. In the latter case the rectangle of intersection is drawn, and all data points lying within the cell are projected orthogonally onto the slicing plane and then drawn as small circles.

The input to ann2fig is a dump file as generated by the dump operation. It is assumed that the file name ends with the suffix ".dmp". The command-line arguments to ann2fig (given below) specify the plane dimensions and the default slicing value and any slicing pairs. The plane dimensions are specified by the -dx and -dy command-line options. By default, the plane dimensions are 0 and 1 for the $x$- and $y$- display dimensions, respectively. The default slicing value is specified by the -sv options. Its default value is 0. Each slicing pair is specified by the -sl option, which is followed by two numbers, the slicing dimension and the slicing value. There may be any number of slicing pairs specified by repeating the -sl option. Other than the plane dimensions, if slicing pairs are not specified for a dimension, then the default slicing value is used instead.

The figure is bounded by the intersection of the bounding box of the kd- or bd-tree with the slicing plane. The size and placement of this bounding box in the final figure is controlled by specifying the $x$- and $y$-offsets of the box (through the -x and -y relative to the upper left corner of the figure (in inches), and the length of the longer side of the box (through the sz option). The box is translated and scaled to match this offset and longest side length before drawing. By default the offsets are each 1 inch, and the size is 5 inches.

There are also command-line arguments to specify the number of fig units per inch (the -upi option), and the radius of the circles used for drawing points (the -ps option, given in fig units). The default units per inch value is 1200, and the default point size value is 10 fig units.

Here is a summary of the command line arguments. Square brackets are used to represent optional arguments, the asterisk ($*$) means that the argument may be repeated zero or more times. The file name is given without the ".dmp" suffix, and the output is written to a file with the same name, but with the suffix ".fig".

ann2fig [-upi *scale*] [-x *xoffset*] [-y *yoffset*] [-sz *size*] [-dx $dim_x$] [-dy *dim_y*]
[-sl *dim value*]* [-ps *pointsize*] *file*

# References

[AM93a] S. Arya and D. M. Mount. Algorithms for fast vector quantization. In J. A. Storer and M. Cohn, editors, *Proc. of DCC '93: Data Compression Conference*, pages 381–390. IEEE Press, 1993.

[AM93b] S. Arya and D. M. Mount. Approximate nearest neighbor queries in fixed dimensions. In *Proc. 4th ACM-SIAM Sympos. Discrete Algorithms*, pages 271–280, 1993.

[AMN+98] S. Arya, D. M. Mount, N. S. Netanyahu, R. Silverman, and A. Wu. An optimal algorithm for approximate nearest neighbor searching. *J. ACM*, 45:891–923, 1998.

[Ben90] J. L. Bentley. K-d trees for semidynamic point sets. In *Proc. 6th Ann. ACM Sympos. Comput. Geom.*, pages 187–197, 1990.

[Boa93] OpenGL Architecture Review Board. *OpenGL Programming Guide*. Addison-Wesley, Reading, MA, 1993.

[Cla97] K. L. Clarkson. Nearest neighbor queries in metric spaces. In *Proc. 29th Annu. ACM Sympos. Theory Comput.*, pages 609–617, 1997.

[FBF77] J. H. Friedman, J. L. Bentley, and R. A. Finkel. An algorithm for finding best matches in logarithmic expected time. *ACM Transactions on Mathematical Software*, 3(3):209–226, 1977.

[Kle97] J. Kleinberg. Two algorithms for nearest-neighbor search in high dimension. In *Proc. 29th Annu. ACM Sympos. Theory Comput.*, pages 599–608, 1997.

[Ove96] M. H. Overmars. Designing the computational geometry algorithms library cgal. In *Proc. 1st ACM Workshop on Appl. Comput. Geom.*, pages 113–119, May 1996.

[PS85] F. P. Preparata and M. I. Shamos. *Computational Geometry: An Introduction*. Springer-Verlag, New York, NY, 1985.

[Spr91] R. L. Sproull. Refinements to nearest-neighbor searching. *Algorithmica*, 6:579–589, 1991.
