# Supplementary material for: Using light to shape chemical gradients for parallel and automated analysis of chemotaxis
Source: Mol Syst Biol. 2015 Apr 23;11(4):804. doi: 10.15252/msb.20156027 (PMC4422560; doi:10.15252/msb.20156027)
Supplement: Supplementary file 15 [file msb0011-0804-sd15.pdf]

Using light to shape chemical gradients for parallel and automated analysis of chemotaxis

Sean R. Collins, Hee Won Yang, Kimberley M. Bonger, Emmanuel G. Guignet, Thomas J. Wandless and Tobias Meyer

Corresponding author: Tobias Meyer, Stanford University School of Medicine

Review timeline:

Submission date:	08 January 2015
Editorial Decision:	23 February 2015
Revision received:	25 March 2015
Accepted:	27 March 2015

Editor: Maria Polychronidou

Transaction Report:

(Note: With the exception of the correction of typographical or spelling errors that could be a source of ambiguity, letters and reports are not edited. The original formatting of letters and referee reports may not be reflected in this compilation.)

1st Editorial Decision 23 February 2015

Thank you again for submitting your work to Molecular Systems Biology. I apologize for the somewhat delayed response which was due to the late arrival of one of the reports. We have now heard back from the three referees who agreed to evaluate your manuscript. As you will see from the reports below, the referees acknowledge that the presented approach seems interesting. However, they raise a series of concerns, which should be carefully addressed in a revision of the manuscript. Since the referees' recommendations are clear, there is no need to repeat the points listed below. The additional follow-up validations suggested by reviewer #2 are not mandatory. However, we would welcome the inclusion of such analyses, if available, since they would indeed enhance the impact of the study.

In line with the comment of reviewer #1 we would like to ask you to provide the Matlab (and other) codes mentioned in the Materials and Methods section. Moreover, as reviewer #1 suggests, the discussion can be extended to include some comments on hits with interesting phenotypes.

We noticed that the study was submitted as a Report. However, since it contains six main figures and considering that we are generally not very strict with the format/character limit, we think that it can be published as an Article, unless there would be any particular reason for which you would prefer to use the Report format. Please do not hesitate to contact me if you would like to discuss this further.

© European Molecular Biology Organization

Reviewer #1:

Understanding machineries leading to cell migration and more specifically non-cell autonomous compound mediated migration is of prime interest and represents a challenging experimental problem as it asks for measuring speed and directionality towards a defined position in perturbed and non perturbed conditions.

Collins and coworkers investigate the mechanisms of chemotaxis and directed migration in mammalian neutrophils model with an elegant approach coupling single-cell tracking, light induced chemo-attractant gradient and gene silencing. The chemo-attractant gradient (ATP or fMLF) is created by a light induced release of the encaged compounds present in the medium. To avoid convection/diffusion-induced movements of the compound and allow the formation of a stable, well-defined chemo-attractant gradient, the medium is made of a mild agarose gel. The authors have then seeded the silenced and control cells in the same well, control and perturbed cells having different fluorescent reporters. Such populations of cells have been tracked and their speed and directionality was evaluated for 285 gene perturbations compared to the control population.

The paper is well written, easy to follow and with clear conclusions. As a general remark the main strength of the work of Collins et al., is to take advantage of the time dimension in a screening context, which will hopefully stimulate other studies using such an approach. Experiments are well-controlled and documented, mixing scrambled and siRNA treated cells in the same well is a very convincing way to control for perturbations.
The relatively low number of genes being clear hits (cofilin, profiling, G-proteins...) is a comforting result and insights into G-protein functions in fMLF sensing, which are more related to directional motility than chemokinesis are of prime interest in the way we understand chemo-attractant-mediated information processing.

No major points.

Minor points.

1. Making available the matlab softwares and codes mentioned in the methods is important. I suggest to make it available on GitHub.

2. Figure labels and abbreviations are often cryptic. Figure 1B, clarify RFU, what is the scale bar? 1D, time unit? Figure 3A, before and after refers to uncaging obviously, but it should be mentioned somewhere.
Figure 4A, not sure this inset is really useful. 4B, showing the actual single cell distribution might be much more convincing. The authors should mention the pValues. 4C, the x axis label "Statistic" might be useless. Figure 5C, this part is really not clear enough, especially the column "Loss of function". Figure 6B, pValues are missing, and also here single cell values distributions might be more suited, however I am fine with histograms.

3. I would recommend not using pixels as a unit in the methods section but rather micrometers.

4. A scheme recapitulating the model the authors propose for the mechanism 0f G-protein involvement in sensing fMLF would be nice.

5. The discussion finishes on a trivial technical statement. I would rather discuss some other hits. From the own authors words, it was surprising to see profilin, cofilin, SSH1 and 2. To me this raises the question of the link between mechanosensing and chemotaxis.

Reviewer #2:

Here Collins et al. describe a novel method to induce chemotaxis by using light to uncage

chemoattractants. A particular advantage to this method is that it can be used to perform high-throughput genetic screens for regulators of chemotaxis. There is every reason to believe the method should also work in small-molecule screens. To demonstrate the utility of this method, the authors then perform a small-scale RNAi screen where they reveal bifurcation of chemotactic signal mediated by G-protein subunits. The authors also use this methodology to explore chemotaxis mediated by ATP.

Certainly the technique is very clever, and because performing high-throughput chemotaxis screens has thus far been challenging, its development represents a significant advance in the area of phenotypic screening especially. Moreover, the authors should be complimented for the rigorousness and care that went into both the validation of the method, and set-up of the RNAi screen.

However I think the manuscript suffers from the fact that the authors have not implemented their methodology to provide new deep insights into chemotaxis, and do not provide any systems-level insights. The screen provides only a small number of (unsurprising?) hits for follow-up. The ATP study also seems to be just the beginning. Taken together, I just don't think this work has sufficient impact on systems biology research to warrant publication in Molecular Systems Biology.

It seems to me this work is far better suited for methods based journals (ie. Nature Methods or Nature Biotechnology), or more cell biology focused journals.

Reviewer #3:

In their manuscript Collins et al describe a new experimental pipeline to genetically screen loss of function mutants in chemotactic granulocytes. They develop a caged fMLP peptide which they incorporate into agarose gels which are layered on top of granulocytes in multi well plates. Upon light exposure the compound is uncaged in a controlled manner, thereby creating chemotactic gradients, which are followed by the cells. Cells are single cell tracked and upon later genetic manipulations internal controls are set up using dual color imaging with labeled nuclei. The method is adequately validated and the authors then go on to screen sets of genes for loss of function phenotypes. They categorize for defects in speed / chemokinesis / angular bias / directed speed and therefore distinguish between effects on basal motility vs. cue-induced motility. They discover and validate a number of expected and unexpected mutants. especially an interesting distinction between G alpha i and beta subunit signaling on induced speed vs. angular bias. Finally they compare fMLP induced chemotaxis to chemotaxis towards uncaged ATP and observe a persistent vs. transient response.
This is very impressive an innovative work. It is very important that this screen uses cells that actually DO chemotax physiologically and hence all findings are of direct relevance. The experimental setting is extremely clever and it has to be appreciated that this is far from easy. Also the accuracy of the methods part is appreciated, where in example the tricks how to layer unpolymerized agarose on top of life granulocytes is faithfully described. The siRNA treatment is far from trivial and builds on an early methods paper of the group.
But this manuscript is not only a technical gold-mine for people working with chemotactic leukocytes. It discovers a number of unexpected mutants that will certainly soon be followed up by this lab and others.
The manuscript is in excellent shape. Of course one could ask numerous follow-up questions on the mutants but I rather see this as a strength of the manuscript as it stands. Hence, I highly recommend publication as it stands.
The only piece of data that I would like to see in the main figures and that is not shown is: what happens to the cells when the gradient is not re-charges, but flattens. How long do the cells memorize the gradient. This would be a useful validation of the uncaring approach and would also be biologically interesting.

© European Molecular Biology Organization

Editorial requests and comments

"*In line with the comment of reviewer #1 we would like to ask you to provide the Matlab (and other) codes mentioned in the Materials and Methods section.*"

- We have made the Matlab code used for the analyses available on Github as requested by Reviewer #1. The code is now freely available for download at https://github.com/srcollins/HT_Chemotaxis_Toolbox.

"*We noticed that the study was submitted as a Report. However, since it contains six main figures and considering that we are generally not very strict with the format/character limit, we think that it can be published as an Article*"

- We would indeed like our study to be published as an Article.

Reviewer #1

Minor points.

1.) *Making available the matlab softwares and codes mentioned in the methods is important. I suggest to make it available on GitHub.*
- We have made the Matlab code used for the analyses available on Github as requested by Reviewer #1. The code is now freely available for download at https://github.com/srcollins/HT_Chemotaxis_Toolbox.

2.) *Figure labels and abbreviations are often cryptic. Figure 1B, clarify RFU, what is the scale bar?*
- We now explain the scale bar in the legend, and we have replaced "RFU" with "Relative Fluorescence Intensity."

1D, time unit?
- We have removed the times from the images in Figures 1B and 1D, and we now instead indicate in the figure legend the times relative to the time of uncaging (in minutes and seconds) that the images represent.

Figure 3A, before and after refers to uncaging obviously, but it should be mentioned somewhere.
- We have adjusted the labels in Figure 3A to state explicitly "Before Uncaging" and "After Uncaging."

Figure 4A, not sure this inset is really useful.
- Although this schematic diagram is not strictly necessary for understanding the experiment, we feel that the visual diagram may help some readers more readily understand our strategy for the in-well use of control cells. For that reason, we have kept the panel.

4B, showing the actual single cell distribution might be much more convincing. The authors should mention the pValues.

- We have added distributions for control and knockdown cells for basal speed, stimulated speed, and angular bias as a new Expanded View Figure (the new Figure E3). As the distribution data is more complicated than the bar graphs, we kept the bar graphs in the main figure for simplicity. The distributions are of statistics from individual movement steps (individual frame to frame step) for individual cells, which we believe reflects the diversity of the cell populations, the stopping and starting behaviors of individual cells, as well as perturbation-dependent differences. We have also added p-values for the normalized cell movement statistics in Figure 4B.

4C, the x axis label "Statistic" might be useless.
- We agree that this label is not necessary, and we have removed it.

Figure 5C, this part is really not clear enough, especially the column "Loss of function".
- We have adjusted the figure panel and the legend to try to make this panel clearer. We have replaced "Loss of function" with "Phenotype:" to indicate that the headings in black text (Basal Speed, Stimulated Speed, Angular Bias, and Chemokinesis) represent different scored phenotypes. We have also expanded the figure legend to explain more explicitly the phenotype groups, as well as the meaning of the text color.

Figure 6B, pValues are missing, and also here single cell values distributions might be more suited, however I am fine with histograms.
- We have added the p-values to the figure, but we have kept the histogram data presentation, as we think it more directly conveys our main findings.

3.) *I would recommend not using pixels as a unit in the methods section but rather micrometers.*
- We now use micrometers as the unit for all details in the methods section, and we provide the corresponding values in pixels for our system in parentheses in case they are also useful.

4.) *A scheme recapitulating the model the authors propose for the mechanism 0f G-protein involvement in sensing fMLF would be nice.*
- We have added a schematic model of our proposed model for G-protein specialization (and the roles of other genes identified in our systematic study) in neutrophil chemotaxis as Figure 6C.

5.) *The discussion finishes on a trivial technical statement. I would rather discuss some other hits. From the own authors words, it was surprising to see profilin, cofilin, SSH1 and 2. To me this raises the question of the link between mechanosensing and chemotaxis.*
- We have now expanded the discussion to discuss some of the more interesting phenotypes identified in the systematic analysis, including a discussion of the potential roles of profilin, cofilin, and SSH1 and 2 in regulating chemokinesis. We mention a possible role of cofilin in integrating mechanical information. These changes are in paragraphs 2, 4, and 5 of the discussion.

Reviewer #2

Reviewer #2 did not raise any specific points, and seemed mostly concerned with whether a methodologically focused paper was suitable for Molecular Systems Biology. In response to this concern, we would like to point out that Molecular Systems Biology does publish articles focused on new methods relevant for systems biology. More importantly, our study in fact provides the first systematic exploration of the functional specialization of a large number of components in eukaryotic chemotaxis signaling systems. Only through systematic analysis of multiple parameters

© European Molecular Biology Organization

is it possible to directly compare the respective roles of the large number of regulators that have been previously implicated in chemotaxis.

<u>Reviewer #3</u>

"*The only piece of data that I would like to see in the main figures and that is not shown is: what happens to the cells when the gradient is not re-charges, but flattens. How long do the cells memorize the gradient. This would be a useful validation of the uncaring approach and would also be biologically interesting.*"

- This is an interesting idea, and we have tried this experiment. However, given the shallow geometry of the uncaging profile, the challenge with the experiment is that the gradient only gradually dissipates after the initial generation. In particular, based on experiments using caged fluorescein, the gradient dissipates slowly by diffusion without the recharging pulses but does not disappear. Thus, the chemoattractant concentration near the center of the field of view gradually decreases over time, and the gradient steepness gradually decreases everywhere. Even so, at the end of our imaging series, there is still a gradient for the cells to follow. The result of the chemotaxis experiments are that cell speed increases after the initial uncaging, but gradually decreases thereafter towards the pre-stimulus basal speed level. The directionality also increases strongly immediately after uncaging and then gradually declines as the gradient dissipates, but the cells do retain some directionality throughout the experiment. It is therefore difficult to interpret how much of this retained directionality may result from cellular memory rather than continued sensing of the remaining gradient. We have pasted in below the results of a representative experiment, but because we think the interpretation is inconclusive, we have not included it as a figure in the manuscript.

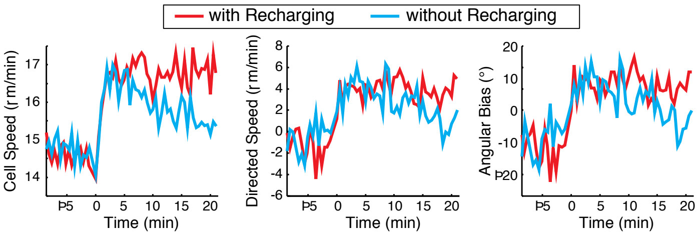

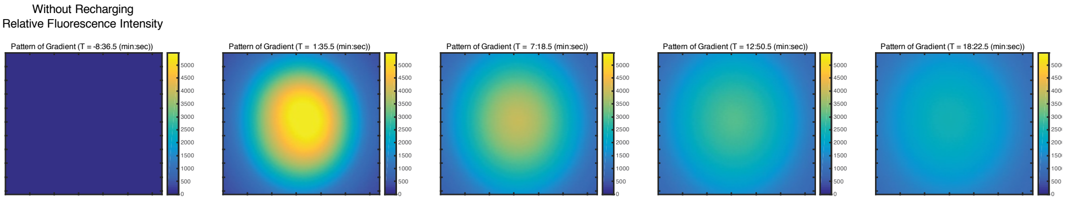

© European Molecular Biology Organization
